# Genome-wide mapping of individual replication fork velocities using nanopore sequencing

**DOI:** 10.1038/s41467-022-31012-0

**Published:** 2022-06-08

**Authors:** Bertrand Theulot, Laurent Lacroix, Jean-Michel Arbona, Gael A. Millot, Etienne Jean, Corinne Cruaud, Jade Pellet, Florence Proux, Magali Hennion, Stefan Engelen, Arnaud Lemainque, Benjamin Audit, Olivier Hyrien, Benoît Le Tallec

**Affiliations:** 1 Institut de Biologie de l’Ecole Normale Supérieure (IBENS), Ecole Normale Supérieure, CNRS, INSERM, Université PSL, 46 rue d’Ulm, F-75005 Paris, France; 2 Sorbonne Université, Collège Doctoral, F-75005 Paris, France; 3 Laboratoire de Biologie et Modélisation de la Cellule, Ecole Normale Supérieure de Lyon, CNRS, UMR5239, INSERM, U1293, Université Claude Bernard Lyon 1, 46 allée d’Italie, F-69364 Lyon, France; 4 Institut Pasteur, Université Paris Cité, Bioinformatics and Biostatistics Hub, F-75015 Paris, France; 5 Genoscope, Institut de biologie François-Jacob, Commissariat à l’Energie Atomique (CEA), Université Paris-Saclay, Evry, France; 6 Université Paris Cité, Epigenetics and Cell Fate, UMR7216, CNRS, Paris, 75013 France; 7 Génomique Métabolique, Genoscope, Institut François Jacob, CEA, CNRS, Univ. Evry, Université Paris-Saclay, 91057 Evry, France; 8 ENSL, CNRS, Laboratoire de physique, F-69342 Lyon, France

**Keywords:** DNA replication, Stalled forks, Nanopores, Machine learning, Genomic instability

## Abstract

Little is known about replication fork velocity variations along eukaryotic genomes, since reference techniques to determine fork speed either provide no sequence information or suffer from low throughput. Here we present NanoForkSpeed, a nanopore sequencing-based method to map and extract the velocity of individual forks detected as tracks of the thymidine analogue bromodeoxyuridine incorporated during a brief pulse-labelling of asynchronously growing cells. NanoForkSpeed retrieves previous *Saccharomyces cerevisiae* mean fork speed estimates (≈2 kb/min) in the BT1 strain exhibiting highly efficient bromodeoxyuridine incorporation and wild-type growth, and precisely quantifies speed changes in cells with altered replisome progression or exposed to hydroxyurea. The positioning of >125,000 fork velocities provides a genome-wide map of fork progression based on individual fork rates, showing a uniform fork speed across yeast chromosomes except for a marked slowdown at known pausing sites.

## Abstract (web summary)

Theulot et al. introduce NanoForkSpeed, a nanopore sequencing-based method to map individual replication fork velocities on entire genomes. NFS shows that fork speed is uniform across yeast chromosomes except for a marked slowdown at pausing sites.

## Introduction

Efficient genome duplication in eukaryotes depends on the proper progression of multiple replication forks. Perturbations of fork movement lead to fork stalling and collapse, generating genomic instability that likely drives cancer development^1^. Despite this pivotal role, the determinants of fork progression are still elusive.

Single-molecule (SM) analyses of DNA replication by DNA fibre autoradiography and its fluorographic evolutions have revealed relatively constant mean fork speeds (1–2 kb/min) in eukaryotic cells together with a broad dispersion of individual fork velocities (0.5–4.0 kb/min) within a given cell line^2–4^ and even within a single cell^5^, suggesting large fluctuations of replication fork speed along eukaryotic genomes. When combined with fluorescence in situ hybridization (FISH) of DNA probes, DNA fibre studies can unveil fork progression within specific regions^6^. In some cases no marked differences between local and genome-wide speeds were observed (e.g., refs. ^6–9^), but slower forks along the pericentromeric and centromeric portions of human chromosomes^10^ and faster forks in long transcribed genes in chicken cells^9^ have been reported. However, only a handful of loci could be analysed given the excessively low throughput of such approaches. To map replication genome-wide, Raghuraman and colleagues determined the mean replication timing (RT) profile of the entire *S. cerevisiae* genome and interpreted the slopes connecting peaks and valleys (i.e., regions of initiation and termination of DNA replication, respectively) as a proxy for local, population-averaged fork velocities, calculating a broad range of speeds depending on genomic location^11^. In sharp contrast, a time-course monitoring of replisome progression found that population-averaged fork velocity is homogeneous throughout yeast chromosomes^12^. It therefore remains unclear whether forks travel at variable or constant speed along eukaryotic genomes.

We have recently developed FORK-seq, a high-throughput, high-resolution, SM-based replication mapping technique relying on the detection by nanopore sequencing of 5-bromo-2’-deoxyuridine (BrdU), a thymidine analogue incorporated in replicating DNA^13^. Here we introduce NanoForkSpeed (NFS), a method capable of positioning, orienting and extracting the velocity of replication forks from BrdU tracks synthesized during a brief pulse-labelling of asynchronously growing cells. NFS allows the determination of the speed of single forks with unprecedented spatial and temporal resolutions, with an unparalleled throughput, and with remarkable simplicity in terms of sample preparation and analysis. Thanks to NFS, we generated in *S. cerevisiae* a genome-wide map of fork progression based on the measurement of individual fork velocities.

## Results

### Detection of BrdU incorporated into replicating DNA

We previously developed the RepNano software to detect BrdU on sequencing reads produced by Oxford Nanopore Technologies (ONT) devices^13^. Here we anchored our BrdU detection process in ONT’s Megalodon program (https://github.com/nanoporetech/megalodon) and estimated BrdU incorporation probability at each thymidine site rather than over 96 bp windows as in RepNano^13^ (see the ‘Methods’ section). Megalodon combines Guppy (ONT) GPU-accelerated basecalling and read mapping to the reference genome into one single step, making it a fast, straightforward and easy-to-use pipeline. Its neural network was trained using nanopore reads of genomic DNA from thymidine-auxotroph MCM869 yeast cells^14^ grown with 11 different proportions of BrdU in the culture medium, from 0 to 100% with increments of 10% (‘Methods’). We first tested the ability of Megalodon to recover from nanopore reads the total BrdU content of these genomic DNA samples determined by mass spectrometry (Supplementary Fig. 1a). RepNano^13^ and other published BrdU basecallers, DNAscent^15^ and DNAscent v2^16^, were also assessed for comparison. Megalodon exhibited the lowest background signal without BrdU and most closely paralleled the perfect correlation line, despite a slight tendency to underestimate BrdU content (Supplementary Fig. 1a). Megalodon also gave the most balanced estimates of BrdU proportion per 1 kb window over the entire range of tested BrdU contents, including the narrowest peaks of null BrdU content corresponding to parental DNA, showing again the lowest background of all basecallers (Supplementary Fig. 1b).

### BT1, a yeast strain with optimised BrdU incorporation and wild-type growth

The budding yeast *S. cerevisiae* lacks a thymidine salvage pathway and is therefore unable to incorporate exogenous thymidine into DNA. Several strategies have been adopted to reconstitute this pathway in vivo in order to render yeasts amenable to DNA labelling with thymidine analogues. They converged towards the combined expression of human equilibrative nucleoside transporter 1 (hENT1), which improves exogenous thymidine uptake, and of Herpes simplex virus thymidine kinase (hsvTK) allowing the conversion of thymidine into thymidine monophosphate (dTMP) (e.g., refs. ^17–19^). Additional inactivation of the thymidylate synthase-encoding *CDC21* gene that is essential for de novo dTMP biosynthesis resulted in strains entirely dependent on external thymidine, or its analogues, for growth^14,20^. We previously analysed the replication of the yeast genome by FORK-seq using one such strain, namely MCM869^13,14^. BrdU incorporation is very high in MCM869 cells due to the absence of competing intracellular thymidine, enabling the detection of replication tracks synthesized during short pulse-chase experiments^13^. However, MCM869 yeasts grew at a reduced rate compared to wild-type (WT) cells even at saturating thymidine concentration (Supplementary Fig. 2a, b). To examine fork progression in WT conditions, we engineered the BT1 strain that retained a functional de novo pathway, and therefore had WT growth properties (Supplementary Fig. 2a–c), while containing codon-optimised *hsvTK* and *hENT1* genes for maximal protein expression and BrdU incorporation in yeast cells. BrdU content profiles of nanopore-sequenced genomic DNA from asynchronously growing BT1 cells pulsed with 100 μM BrdU for 2 min followed by a 20 min chase with 1 mM thymidine showed that BrdU incorporation was of comparable efficiency as in MCM869 cells (Supplementary Fig. 2d). In contrast, replication tracks were hardly detectable on pulse-labelled DNA from thymidine-prototroph, BrdU-incorporating strains constructed using currently available hsvTK and hENT1 integrative vectors^17,19^ (BT2 and BT3 strains, Supplementary Fig. 2d). BT1 thus constitutes a potent tool to examine fork progression in physiologically relevant conditions.

### Fork orientation and fork speed measurement by NFS

**Fig. 1:**
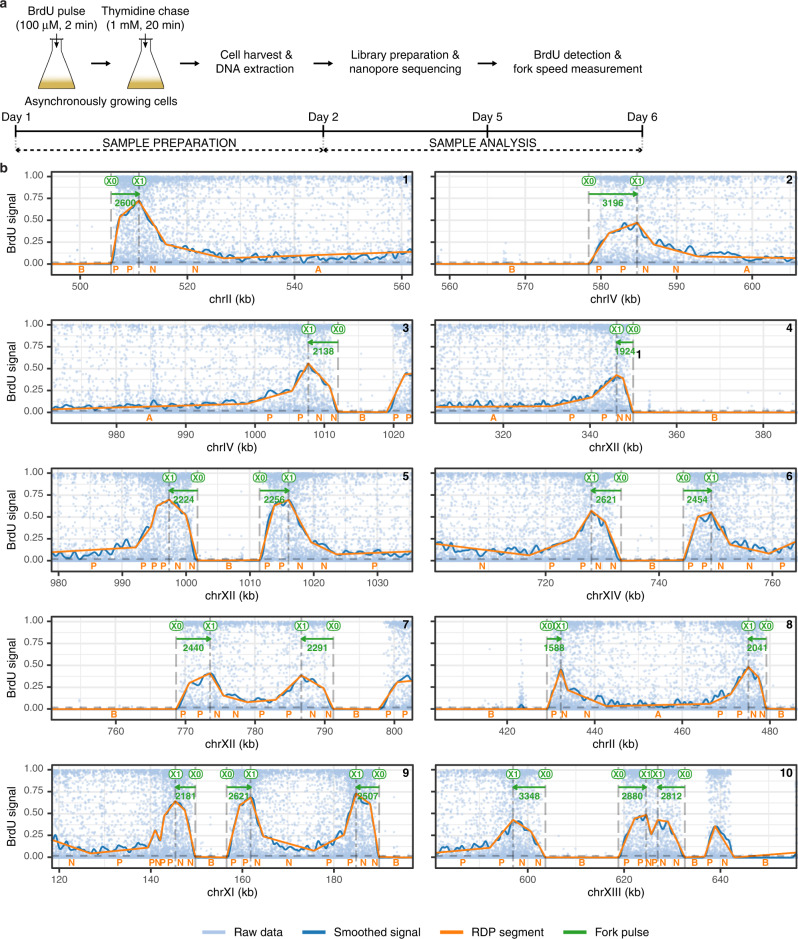
Replication fork speed measurement procedure by NFS. **a** Scheme of the protocol for BrdU pulse-labelling of DNA replication in BT1 cells. The usual timeline is indicated. **b** BrdU content profiles of nanopore sequencing reads of genomic DNA from pulse-labelled BT1 cells processed by NFS. Panels show typical replication signals, namely rightward, leftward, diverging and converging forks. Light blue dots, raw data from Megalodon (dots represent the probability of BrdU at each thymidine position); blue curve, smoothed signal; orange lines, segments resulting from the piecewise linear simplification method using the Ramer-Douglas-Peucker algorithm (RDP) to detect and orient BrdU tracks (B, flat segments with background BrdU level; A, flat segments with a BrdU level above background; P, segments with a positive slope; N, segments with a negative slope); X0, estimated position of the start of BrdU incorporation; X1, estimated position of the start of the thymidine chase; green arrow, fork direction, with fork velocity (bp/min, in green) indicated below.

Typical BrdU signals in sequencing reads of genomic DNA from pulse-labelled BT1 cells (Fig. 1a) displayed an asymmetrical shape, consisting of a steep ascending slope starting from a segment of null BrdU content (BrdU signal increasing from 0 to ≈0.5), followed by a shallower decreasing slope (from ≈0.5 to ≈0.1) (Fig. 1b). These signals resembled those from MCM869 cells (Supplementary Fig. 2d), previously demonstrated to correspond to elongating replication forks, with the steep and shallow slopes reflecting BrdU incorporation during the pulse and the chase, respectively, and the signal asymmetry revealing fork direction^13^. Since fork speed can be measured as the length of the labelled track divided by the corresponding labelling time, we developed a pipeline named NFS to capture the track length synthesized during the BrdU pulse, that is the section between the starting and ending points of the steep slope. A piecewise linear simplification method first converted reads into a sequence of segments classified into 4 categories based on their slope and mean BrdU signal: (i) flat segments with background BrdU level (B); (ii) flat segments with a BrdU level above background (A); (iii) segments with a positive slope (P); and (iv) segments with a negative slope (N) (Fig. 1b). We then identified two patterns for elongating forks depending on their direction. Rightward forks were preceded by a B segment which replicated before the BrdU pulse, then consisted in one or successive P segments, occasionally interrupted by A segments owing to noise, and followed by at least one N segment corresponding to DNA replicated during the chase (Fig. 1b, reads 1, 2). Consequently, we used a regular expression procedure to search for the “BP(P|A)*N+” pattern. Leftward forks were recognized with the symmetrical pattern “P+(N|A)*NB” (Fig. 1b, reads 3, 4). This procedure excluded both incomplete replication tracks (Fig. 1b, read 3, position 1020 kb, and read 7, position 800 kb) and symmetrical signals due to pairs of forks initiated after the start of the pulse or terminated before the end of the pulse, for which the actual labelling time could not be precisely estimated (Fig. 1b, read 10, position 640 kb). The precision of track detection and orientation by NFS was confirmed by (i) a virtually null false-positive rate (≈50 mapped forks per 10 Gb of DNA with no BrdU labelling versus ≈15,000 mapped forks per 10 Gb of pulse-labelled DNA; Supplementary Data 1); (ii) the remarkable similarity of BT1 replication fork directionality (RFD) profile aggregating oriented replication tracks detected by NFS with MCM869 RFD profiles computed by FORK-seq or Okazaki fragment sequencing^13^ (Spearman’s pairwise correlation coefficients of 0.83 and 0.88, respectively, Supplementary Fig. 3a, b) and (iii) the spatial coincidence between known yeast origins^21^, upward slopes (i.e., initiation regions) on BT1 RFD profile and individual initiation sites defined as the midpoints between diverging forks (Supplementary Fig. 3a, c).

For every read, NFS yielded the coordinates of each of the B/P/A/N segments decomposing replication tracks. The length of DNA replicated during the BrdU pulse was then readily computed as the distance between the B/P (X0, start of BrdU incorporation) and (P|A)/N transitions (X1, start of the thymidine chase) for rightward forks and between N/B (X0) and P/(A|N) (X1) transitions for leftward forks (Fig. 1b). Fork velocity was subsequently calculated as the ratio between track length and BrdU pulse duration. The latter was set to 2 min, which was sufficient for detection of replication signals while brief enough to maximize the probability of obtaining complete tracks along nanopore reads averaging ≈15 kb in length. We first conducted a pilot experiment in which BT1 cells were pulsed with BrdU doses ranging from 10 μM to 1 mM and chased with a ten-fold excess of thymidine to determine the optimal labelling conditions. As anticipated, the maximal BrdU signal amplitude rose with increasing BrdU concentrations, whereas similar velocities were computed regardless of the BrdU dose (Supplementary Fig. 4). This indicated that our pipeline was functional for a broad range of signal amplitudes and that fork speed measurement by NFS was independent of BrdU concentration in the tested range. However, in line with reports that high doses of BrdU are toxic for TK-expressing *S. cerevisiae* strains^20,22^, we found that BrdU concentrations over 100 μM both slowed down BT1 S phase and triggered checkpoint activation (Supplementary Fig. 5). Although these doses did not visibly impact fork speed (Supplementary Fig. 4), probably because of the very limited exposure to BrdU during the 2 min pulse, we opted for a pulse concentration of 100 μM BrdU.

### Validation of fork speed measurement by NFS

**Fig. 2:**
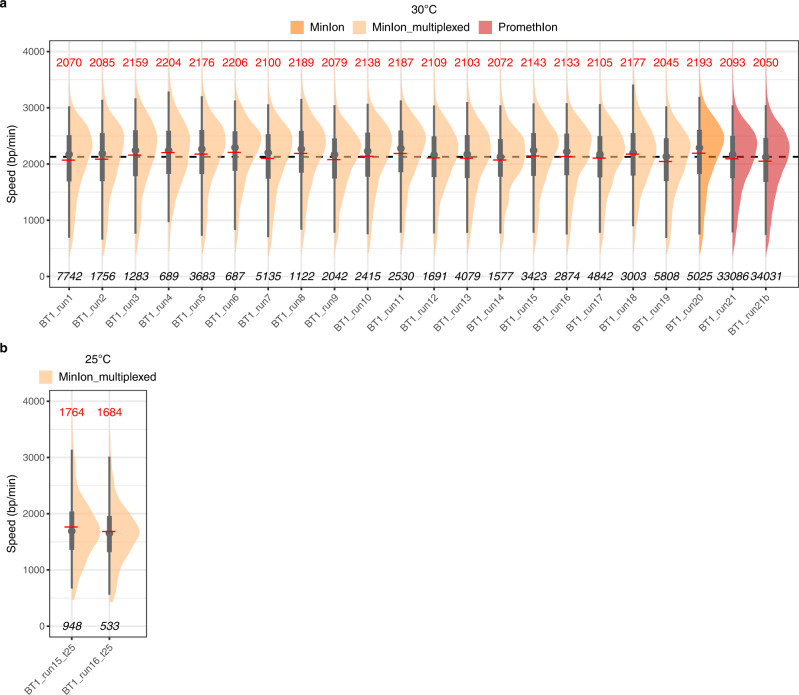
Measurement of replication fork speed in yeast by NFS. **a**, **b** Replication fork speed in BT1 cells grown at 30 °C (**a**) or 25 °C (**b**). Half-eye plots of individual fork velocities detected by NFS on sequencing reads from MinION, multiplexed MinION or PromethION runs performed on pulse-labelled DNA from independent BT1 cell cultures (the two PromethION samples are technical replicates). Red line, mean speed, value indicated in red on top; grey dot, median speed; thick and thin grey vertical lines, 50 and 95% intervals, respectively; bottom, number of individual fork speed measurements; dotted line in **a**, average fork speed of all samples (2128 bp/min). The name of the sequencing run is indicated below each plot; detailed run information is presented in Supplementary Data 1.

We performed multiple independent pulse-labelling experiments using the aforementioned conditions (Fig. 2a). Thousands (tens of thousands) of individual measurements were typically collected in a single run using the ONT MinION (PromethION) device. All experiments yielded similar fork speed distributions and mean velocities between 2045 and 2206 bp/min (average of 2128 bp/min, Fig. 2a), emphasizing the reproducibility of our analysis. Above all, these values are in excellent agreement with previous estimates of ≈2 kb/min in *S. cerevisiae* cells grown at 30 °C^23–26^. NFS also found that the mean fork speed was 1.7 kb/min in cells grown at 25 °C, which again agreed very well with the average progression rate of 1.6 kb/min determined at this temperature in a preceding study^12^ (Fig. 2b).

### Using simulated replication forks to evaluate NFS accuracy

**Fig. 3:**
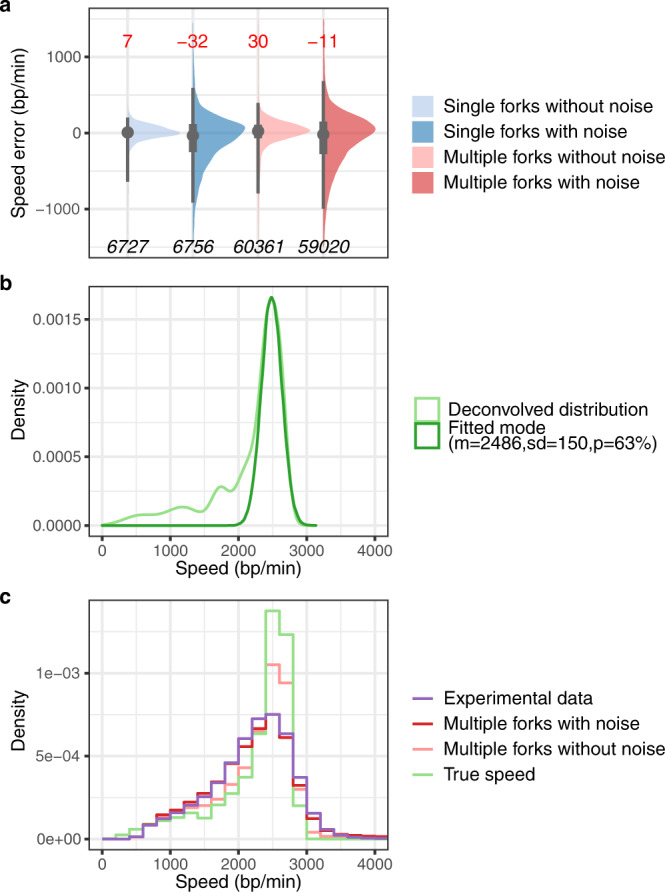
Evaluation of NFS accuracy and estimation of the true fork speed distribution in yeast using simulated replication forks. **a** Half-eye plots showing the distribution of measurement errors made by NFS on simulated forks. Fork velocities were determined by NFS on simulated reads containing either a single or multiple forks of known speed, with or without noise, and differences between NFS measurements and true speeds were computed (see ‘Methods’). Grey dot, median speed error, value indicated in red on top; thick and thin grey vertical lines, 50% and 95% intervals, respectively; bottom, number of measurements. **b** Deconvolved distribution of individual fork speeds in yeast (light green curve) and Gaussian distribution fitting its main peak (green curve). m, mean speed (bp/min); sd, standard deviation (bp/min); p, percentage (correction factor to fit the Gaussian distribution to the deconvolved distribution). See text and ‘Methods’ for details. **c** Histograms showing fork speed distribution on 100,000 reads with one or multiple forks simulated according to the deconvolved true fork speed distribution (True Speed, light green), fork speed distribution after NFS processing of these reads without (pink) or with (red) noise, and the experimental distribution of >125,000 individual fork velocities from BT1 cells at 30 °C (purple).

To further gauge NFS, we generated in silico reads with BrdU signals mimicking replication forks travelling at a defined velocity (‘Methods’) and tested NFS ability to recover these velocities. Simulated forks had similar amplitude range, signal dynamics and noise as those from authentic reads of 2-min, 100 μM BrdU pulse-labelled BT1 DNA (Supplementary Fig. 6). In addition, they displayed the same speed distribution as that of real sequencing data to allow the comparison of our estimates after NFS processing to a known ground truth under the conditions of an actual experiment (‘Methods’). NFS measurements were extremely precise on simulated reads bearing one or multiple forks in the absence of signal noise (median error of 7 and 30 bp/min and interquartile range (IQR) of 123 and 186 bp/min for single and multiple forks, respectively; Fig. 3a). Importantly, although speed error distributions broadened when NFS was confronted to simulated forks with noise closely resembling experimental data (IQR of 371 and 438 bp/min for single and multiple forks, respectively; Fig. 3a), the median error remained remarkably low (−32 and −11 bp/min for single and multiple forks, respectively; Fig. 3a), demonstrating that the overall evaluation of fork velocity by NFS was extremely accurate. We noted that NFS tended to overestimate and underestimate slow and fast forks, respectively, and that the dispersion of NFS measurements increased with fork speed (Supplementary Fig. 7). However, the median speed error remained below 10% relative to theoretical velocities in the 1000 to 3000 bp/min range, which encompasses the vast majority of physiological fork rates (Supplementary Fig. 7).

### Estimating the true fork speed distribution in yeast

The distribution of fork velocities determined by NFS in BT1 cells is an approximation of the genuine distribution of individual fork speeds in yeast owing to signal noise and measurement errors. Since we could characterize how NFS responded to fork velocities of known value thanks to simulated reads, we adopted a deconvolution strategy to predict the original fork speed distribution from our >125,000 experimental measurements at 30 °C (‘Methods’). Interestingly, the main peak of our estimate of the true speed distribution consisted of values in the 2486 ± 150 bp/min range that accounted for two-thirds of all fork velocities (Fig. 3b), suggesting a globally low dispersion of individual fork speeds in *S. cerevisiae*. Finally, to evaluate the deconvolution procedure, we generated 100,000 reads bearing forks simulated on the basis of the deconvolved true fork speed distribution and analysed them with NFS; the obtained distribution closely approximated the experimental one (Fig. 3c), validating our approach.

### NFS retrieves expected shifts in fork speed

**Fig. 4:**
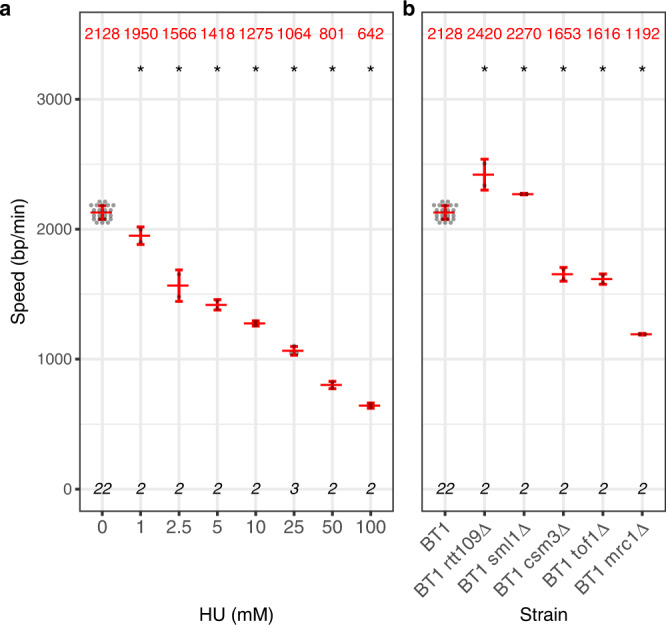
Measurement of replication fork speed in yeast by NFS in conditions of altered replisome progression. **a** Replication fork speed in BT1 cells grown in the presence of increasing concentrations of hydroxyurea (HU). **b** Replication fork speed in mutant strains with altered fork progression. **a**, **b** Grey dot, mean fork speed of a sample; red centre line, average value of all mean fork speeds of the corresponding category, indicated in red on top; red whiskers, standard deviation; bottom, number of samples. Two-sided contrast comparisons between untreated BT1 cells and each HU concentration in **a** and between BT1 cells and each mutant in **b** are indicated by a star (*p* ≤ 0.05) or n.s. (not significant). Statistical analyses are detailed in Supplementary Table 2.

We next investigated if NFS was able to detect changes in replication fork velocity in conditions known to alter fork progression (Fig. 4). We first performed pulse-chase experiments in cells exposed to hydroxyurea (HU), a commonly used drug inducing replication fork slowdown. As expected, we observed a gradual decrease in fork speed with increasing HU concentrations (Fig. 4a). We could detect fork slowdown induced by doses as low as 1 mM HU, indicating that NFS is a very sensitive tool to reveal replication stress. We then measured fork velocity in cells with mutations impacting replisome progression. We were able to recover the previously reported decrease in fork speed in the absence of Mrc1^25,27–30^, Tof1^28,30^ and Csm3^30^ (Fig. 4b). Interestingly, fork speed was equally reduced in *tof1Δ* and *csm3Δ* cells, consistent with the fact that these proteins form a complex at the front of the replisome^31,32^. Moreover, fork progression was affected to a lesser extent in those mutants than without Mrc1, as already described^29,30,33^. NFS also detected the acceleration of replication forks observed in the absence of Rtt109 acetyltransferase^26^, as well as in *sml1Δ* mutant exhibiting increased dNTP pools^34^ (Fig. 4b). Altogether, these results demonstrate that NFS can precisely quantify fork speed variations both in physiological and perturbed conditions.

### Genome-wide mapping of replication fork progression

**Fig. 5:**
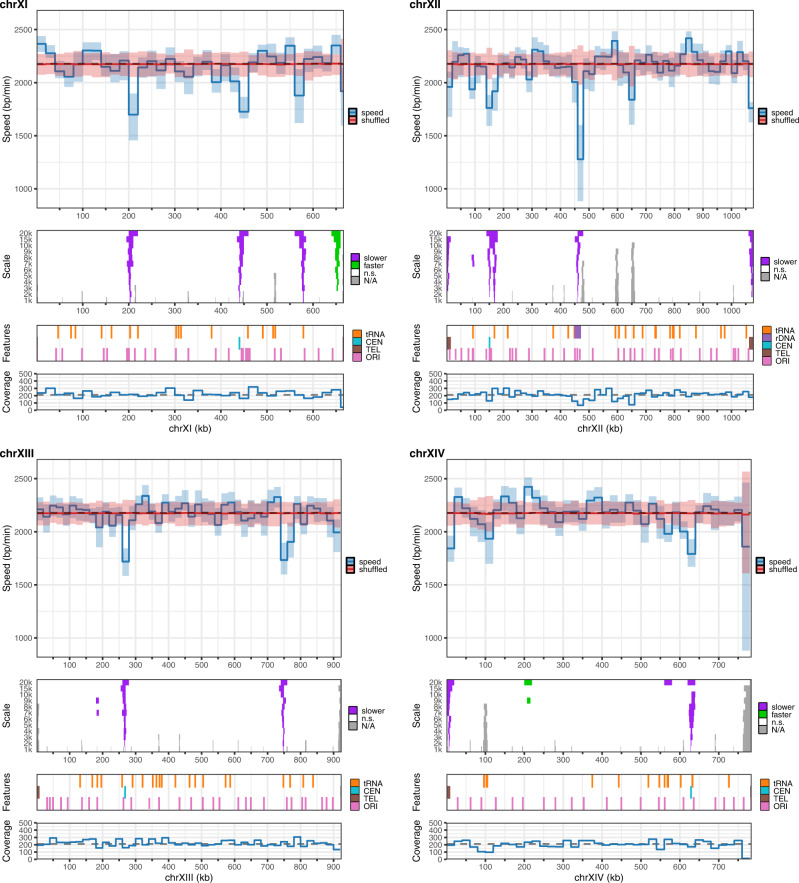
Replication fork progression map of yeast chromosomes. Shown are chromosomes XI to XIV. Panels from top to bottom: (1) median of experimental fork speeds (blue line) with 98% confidence interval of the median (light blue) and median of reshuffled speeds (red line) with 98% confidence interval of the median (light red) computed in 20 kb windows (dotted line, median fork speed in the whole genome); (2) results of Mann–Whitney-Wilcoxon tests with Holm correction (one-sided) performed along the chromosome to compare the speed distribution in a given window of a given width (1, 2, 3, 4, 5, 6, 7, 8, 9, 10, 15 and 20 kb) to the speed distribution on the whole genome (purple, regions of lower fork speed; green, regions of higher fork speed; white, n.s., not significant; statistical significance was set to *p* < 0.01; grey, N/A, not applicable, regions with no fork); (3) position of selected genomic features (CEN, centromere; TEL, telomeres; ORI, known *S. cerevisiae* replication origins from ref. ^21^); (4) coverage of individual replication fork velocities (dotted line, median coverage of the genome). N/A peaks in panel 2 and droughts in coverage in panel 4 essentially map to the position of active origins since fork pairs at initiation sites cannot be resolved by our analysis (see text for details).

**Fig. 6:**
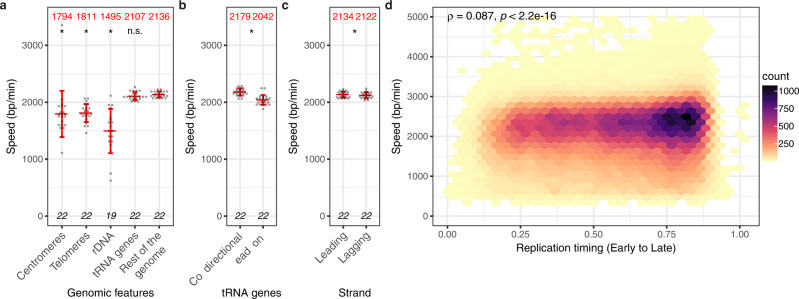
Analysis of replication fork progression in the yeast genome. **a** Replication fork speed at selected genomic features. Please note that “telomeres” correspond to subtelomeric sequences and do not comprise the terminal stretch of telomeric repeats. **b** Replication fork speed at tRNA genes with a direction of transcription in the same (Co-directional, 129 genes) or opposite (Head-on, 115 genes) orientation to the main direction of fork progression determined using BT1 RFD profile (see ‘Methods’ and Supplementary Fig. 3). **c** Replication fork speed on the leading and lagging strands. **a**–**c** Grey dot, mean speed of forks overlapping the corresponding DNA element (**a**, **b**) or progressing on the leading or lagging strand (**c**) in a sample; red centre line, average value of all mean fork speeds of the corresponding category, indicated in red on top; red whiskers, standard deviation; bottom, number of samples (*n*_rDNA_ = 19 since no fork overlapped the rDNA locus in 3 samples). Two-sided contrast comparisons between a given genomic feature and the rest of the genome in **a**, between co-directional and head-on tRNA genes in **b** and between the leading and lagging strands in **c** are indicated by a star (*p* ≤ 0.05) or n.s. (not significant). Statistical analyses are detailed in Supplementary Table 2. **d** Fork speed versus replication timing (data from ref. ^42^ normalized between 0 and 1 corresponding to the start and end of S phase, respectively) plotted as a 2D density plot using hexagonal bins. Coefficient (*ρ*) and *p*-value of Spearman’s correlation test (two-sided) between fork speed and replication timing are indicated.

NFS allowed us to generate a genome-wide map of replication fork progression based on individual fork velocities (Fig. 5 and Supplementary Fig. 8). In order to distinguish meaningful changes in local fork speed distribution, the experimental map was superimposed to a randomized map where fork locations remained unchanged but velocity values were shuffled. This showed that fork movement was largely uniform across the yeast genome, in agreement with the results obtained by Sekedat and colleagues^12^, although several loci clearly stood out. We next performed a multi-scale statistical analysis to precisely identify regions of significantly lower or higher fork speed than the bulk genome along with the underlying factors likely responsible for the observed alterations. We detected several regions of fork slowdown but only one locus, located between the *FLO10* and *NFT1* genes on chromosome XI, showed fork acceleration (Fig. 5 and Supplementary Fig. 9). Salient features inside slow regions narrowed down to 1 kb included centromeres, telomeres, tRNA genes, the rDNA locus and the HML silent origin cluster (Fig. 5 and Supplementary Fig. 8), all known impediments to replication forks^35,36^. Targeted analysis of forks overlapping these DNA elements confirmed that centromeres and telomeres quite consistently exhibited a reduced fork speed (Fig. 6a and Supplementary Fig. 10) and that fork slowdown at the rDNA locus occurred in the vicinity of the replication fork barrier (RFB), as anticipated (Supplementary Fig. 11). Fork progression was globally slower at tRNA genes having a direction of transcription opposing that of forks (Fig. 6b), in agreement with tRNA genes being polar obstacles to replication^35–37^, although speed was markedly reduced only at a small subset of tRNA genes (Supplementary Fig. 12). We speculate that those exhibit a particularly strong binding of TFIIIB transcription factor, considered as the main impediment to fork movement at tRNA genes^38^. Thanks to the mapping of oriented tracks to Watson or Crick strands, we could distinguish fork progression on the leading and lagging strands. We found that DNA synthesis proceeds significantly faster on the leading strand (Fig. 6c), although the very weak difference in speed with the lagging strand (12 bp/min) is possibly devoid of biological significance. Finally, as it remains unclear whether fork velocity changes during S phase in eukaryotes^2,3,8,39–41^, we compared the speed of replication forks with the timing of the region they are replicating^42^, noting that forks appear to slightly accelerate during S phase in yeast (Fig. 6d).

## Discussion

Under conditions of BrdU incorporation that do not perturb DNA replication, NFS recovers former estimations of fork speed in yeast in a WT-like growing strain and in mutants known to have altered fork progression, with an excellent reproducibility between biological replicates. This validates our measurement procedure and emphasizes the robustness of our method. Moreover, NFS is remarkably efficient in detecting replication fork slowdown, from the slightest to major decelerations, making it a suitable tool to detect replication stress. NFS accuracy has been further verified on simulated forks of known speed, which also offered the opportunity to recreate the presumed true fork velocity distribution in *S. cerevisiae* from the experimental distribution that is inevitably altered by NFS measurement errors, however small they may be. Our results suggest that the majority of fork speeds are narrowly distributed in yeast (two-thirds within 2500 ± 150 bp/min), in apparent contrast with the large dispersion of individual fork velocities observed by conventional SM methods in eukaryotes^2–4^. However, it is important to note that although a 10-fold difference between the fastest and slowest forks is commonly found in standard DNA fibre analysis of a given cell line, most fork rates actually fall within less than twofold of the median velocity^4^. For instance, half of the forks travel within ±20% of the 1.5 kb/min median speed in chicken DT40 cells^9^. Extreme values may reflect rare events or may be, at least in part, artefactual. For instance, exceptionally high velocities may originate from the fusion of forks emanating from adjacent origins that fired during the labelling pulse, which may go unnoticed even when using a robust analysis methodology^4^. In that regard, the use of a much shorter labelling pulse in our conditions compared to standard SM techniques minimizes the probability that BrdU tracks result from the activity of more than one fork.

In agreement with a low dispersion of fork speeds in *S. cerevisiae*, the mapping of individual fork velocities shows that the rate of fork progression does not significantly change across the yeast genome except at previously known fork pausing sites^35,36^, plus at a single locus situated between *FLO10* and *NFT1* genes where forks appear to move faster for reasons that remain to be determined. We have not attempted here to verify whether low fork speeds around pausing sites result from transient stalling rather than uniformly slow progression, but the former seems likely, especially since fork slowdown at the rDNA locus mapped to the ribosomal RFB pausing site. Our observations of a consistent velocity across the yeast genome support several earlier studies^12,24^ and are in line with a very recent nanopore sequencing-based analysis of excised full-length replicons concluding that fork progression is remarkably homogeneous along *S. cerevisiae* chromosomes^43^, but they are at odds with those of Raghuraman and colleagues who interpreted variations in RT profile slopes along different parts of the genome as fluctuations in fork speed^11^. However, it has since been demonstrated that RT slopes depend not only on fork speed but also on the local proportion of rightward and leftward forks in the cell population^8,44,45^. Our results suggest that RT profile slope variations observed in that pioneering study predominantly if not exclusively stem from variations in the proportion of rightward and leftward forks.

Determining fork speed is mandatory in circumstances known or speculated to influence replication dynamics. Existing methods include DNA fibre analysis^2–4,46^, hydrodynamic techniques^2,3^, slope computation from RT profiles (e.g., refs. ^8,11,25,26,47–50^), mathematical modelling of DNA replication (e.g., refs. ^24,44^) and time-resolved (i) dense-isotope substitution experiments (for instance, see ref. ^51^), (ii) two-dimensional gels^27^, (iii) chromatin immunoprecipitation of replisome components^12,23,26,27^ or (iv) isolation of BrdU- or 5-ethynyl-2’-deoxyuridine-labelled DNA^52,53^. All these approaches are demanding and difficult to implement, and none of them allows the mapping of individual fork velocities on entire genomes. Not only does NFS have this ability, but it also combines a straightforward protocol with a ready-to-use analytical pipeline (available on GitHub at https://github.com/LacroixLaurent/NanoForkSpeed) enabling an automated measurement of fork speed. NFS should advantageously replace DNA fibre analysis as the “gold standard” method to determine fork velocity as it surpasses it in simplicity, rapidity (estimation of fork speed in 6 days for NFS versus weeks or months for DNA combing or spreading especially when combined with FISH probe detection), number of measurements (thousands of values versus a few tens to a few hundreds) as well as in spatial (precision approaching the nucleotide resolution of sequencing versus precision limited to ≈1 kb by the resolution of optical microscopes) and temporal (fork speed averaged over 2 min versus typically 20 min) resolutions. Estimation of fork velocity by NFS is certainly applicable to BrdU pulse-labelled DNA from other eukaryotes, notably mammalian cells, although a higher sequencing depth will be required to obtain a comparable coverage. The rapid evolution of nanopore sequencing technologies offering increased throughputs at reduced costs will contribute to overcome this limitation. Meanwhile, the simultaneous processing of multiplexed samples should facilitate the screening of knockouts libraries, particularly in yeast, to uncover new factors involved in replisome progression and broaden our understanding of eukaryotic DNA replication.

## Methods

### Yeast strains and growth conditions

All strains used in this study are W303 derivatives and are listed in Supplementary Table 1. Standard yeast genetic techniques and media were used^54^. Cells were grown at 30 °C in YPD medium (MP Biomedicals #114001032) unless stated otherwise. BT1 strain was obtained by integrating at the *ura3-1* locus of W303 cells StuI-linearized pBL-hsvTK_CO_-hENT1_CO_ plasmid (Supplementary Table 1), a modified version of the p306-BrdU-Inc vector^17^ in which both the *hsvTK* and *hENT1* genes were replaced by versions that had been codon-optimized for expression in yeast. *hsvTK* was codon-optimized for yeast using JCat^55^ and synthesized by GeneScript; codon-optimized *hENT1*^19,56^ was a generous gift from E. Schwob (IGMM, Montpellier, France). Detailed cloning procedures are available upon request. The BT2 strain was obtained by reintroducing the WT *CDC21* allele encoding the thymidylate synthase into the MCM869 strain^14^ by crossing. The BT3 strain was obtained by inserting the regular p306-BrdU-Inc plasmid^17^ purchased from Addgene at the *ura3-1* locus of W303 cells.

### Growth curve and doubling time

Yeasts were grown overnight in YPD (supplemented with 100 μM thymidine for MCM869 cells), diluted in fresh medium (supplemented with 100 μM thymidine for MCM869 cells) at an optical density at 600 nm (OD_600_) ≈ 0.1 and grown for 6 h. OD_600_ was measured every 2 h. Doubling times (*T*) were estimated on the basis of the growth curves based on the formula *T* = [culture duration*log(2)]/[log(final OD_600_) − log(initial OD_600_)].

### Analysis of S phase progression by fluorescence-activated cell sorting (FACS)

Exponentially growing cells were synchronized in G1 by addition of 0.2 μM α-factor (Sigma #T6901) for 3 h then washed and resuspended in fresh, prewarmed YPD medium containing 50 μg.mL^−1^ pronase (Millipore #53702) to release them into the cell cycle. In the experiment examining the impact of BrdU on S phase progression, BrdU was added 15 min after cell release. Aliquots were taken at regular time intervals and fixed in ethanol. Fixed cells were washed with 50 mM sodium citrate pH 7.4, incubated in sodium citrate buffer supplemented with 0.25 mg.mL^−1^ RNAse A for 1 h at 50 °C, added with 2 mg.mL^−1^ proteinase K and incubated for one additional hour. DNA was counterstained overnight with 1 μM SYTOX Green (Invitrogen #S7020). Samples were analysed using a Beckman Coulter CytoFLEX LX flow cytometer. Data were collected using CytExpert v2.4.0.28 and analysed using FlowJo v10.7.1. Gating strategy is illustrated in Supplementary Fig. 13.

### Rad53 immunoblot analysis

Total protein extracts were obtained by trichloroacetic acid (TCA) precipitation, separated by SDS-PAGE on a 7.5% gel and transferred to a nitrocellulose membrane. Rad53 immunoblot was performed with rabbit anti-Rad53 antibody at 1:10,000 (Abcam #104232, batch GR3353005-2), using HRP-conjugated anti-rabbit (Promega #W401B) at 1:5000 as secondary antibody. Detection was performed with SuperSignal West Pico (Thermo Scientific #34078) chemiluminescent reagents. ImageQuant LAS 4000 mini (GE Healthcare, software version 1.3) was used for imaging.

### Samples used for neural network training

Neural network training was performed using nanopore-sequenced genomic DNA displaying variable BrdU substitution rates extracted from the thymidine-auxotroph MCM869 strain. For that purpose, MCM869 cells were grown overnight in synthetic complete medium (Dropout Base medium with Complete Supplement Mixture, MP Biomedicals #114025032 and #114500012) with 100 μM thymidine, washed twice to remove thymidine, transferred at OD_600_ ≈ 0.1 into fresh synthetic complete medium supplemented with various mixtures of BrdU and thymidine (0:100; 10:90; 20:80; 30:70; 40:60; 50:50; 60:40; 70:30; 80:20; 90:10 and 100:0) and grown for 24 h. Genomic DNA was isolated by zymolyase, RNAse A, and proteinase K digestion using Genomic DNA Buffer Set (Qiagen #19060) and Qiagen Genomic-tips 20/G (Qiagen #10223) according to the manufacturer’s instructions and subsequently subjected to nanopore sequencing.

### Mass spectrometry

LC-MS/MS was performed on the 11 samples prepared for neural network training (see above) using a TSQ Quantiva triple quadrupole mass spectrometer (Thermo Scientific) coupled to an UltiMate 3000 XRS HPLC system (Dionex, Thermo Scientific) as described in ref. ^13^. Samples were analysed in technical duplicates using the software TraceFinder (Thermo Scientific, version 5.1).

### Pulse-chase labelling

In most experiments, BT1 cells and its derivatives as well as BT2 and BT3 strains were grown overnight in YPD, diluted in fresh medium at an OD_600_ ≈ 0.1 and pulsed after 3 doublings (OD_600_ ≈ 0.8) with 100 μM BrdU for 2 min followed by a 20 min incubation with 1 mM thymidine. For the experiment carried out with different BrdU concentrations, BT1 cells were pulsed in the same conditions as above with either 10, 25, 50, 100, 250, 500, 750 μM or 1 mM BrdU and chased with a ten-fold excess of thymidine. For experiments in the presence of HU, cells were grown for 2 doublings (OD_600_ ≈ 0.4) and treated for 1 h with HU prior to BrdU pulse-labelling. MCM869 cells were cultured overnight in YPD supplemented with 100 μM thymidine, diluted in fresh YPD with thymidine at an OD_600_ ≈ 0.1 and grown to OD_600_ ≈ 0.8 before being washed twice with YPD to remove thymidine, transferred to YPD for 30 min, pulsed with 100 μM BrdU for 2 min and chased with 1 mM thymidine for 45 min. Cells were pelleted after the thymidine chase, washed with water before DNA extraction as in ref. ^57^.

### Library preparation and data acquisition

All samples were sequenced using R9.4.1 chemistry flow cells from Oxford Nanopore Technology (ONT). MinION and GridION sequencing libraries were prepared using ligation sequencing kits SQK-LSK108 or SQK-LSK109 (ONT) in combination with ONT EXP-NBD103 Native Barcoding kit or ONT EXP-NBD104 Native Barcoding Expansion 1–12 pack in case of multiplexing according to ONT protocols with the modifications presented in ref. ^57^. PromethION sequencing libraries were prepared using the ultra-long DNA sequencing kit SQK-ULK001 (ONT) according to ONT protocols. Data were acquired using MinKNOW (ONT, MinKNOW Core versions 1.14.1 to 4.4.13, Supplementary Data 1) with default parameters. Demultiplexing of barcoded nanopore reads was performed as described in ref. ^57^.

### Training of the BrdU basecalling model

Our model was built in three successive steps. First, we modified the architecture of RepNano convolutional neural network^13^ in order to obtain a nucleotide resolution for BrdU detection, creating RepNano v2. We next performed a first training of our model with ONT’s Taiyaki from the Megalodon program (version 2.2.9 downloaded from https://nanoporetech.github.io/megalodon/, Guppy version 4.4.1) using RepNano v2 outputs, then trained our model a second time using the outputs of the first training and adding specific false positive BrdU signals to the training dataset in order to reduce background and false-positive signals.

#### RepNano v2 architecture

We used two convolutional layers with kernel size 7 and filter size 32 with no padding followed by a long short-term memory (LSTM) layer with 32 hidden units that output a vector of the same length as the input, then a convolutional layer with kernel size 1 and filter size 1 and finally an averaging layer outputting the BrdU percent of the segment. The initial length of the input vector was 112 so that after two convolutions without padding the final length was 100. The LSTM layer also produced a 100 × 32 vector and the last convolutional layer produced a 100 × 1 vector that was averaged and compared to the expected output of BrdU content via a logcosh loss. We extracted the vector from the last convolutional layer to basecall BrdU at the nucleotide level.

#### RepNano v2 training

RepNano v2 was trained using nanopore reads of the 11 genomic DNA samples with various BrdU substitution rates described above (BrdU contents measured by mass spectrometry of 0, 9.4, 16.6, 27.9, 35.1, 46.1, 54.8, 59, 72.6, 78.8 and 80.3%). For each of the 11 samples, composed of a mix of substituted and unsubstituted reads (corresponding to parental DNA), 400 reads were used for the learning. To separate substituted reads from unsubstituted ones, we first ran two training cycles with a neural network made of only three convolutional layers (no LSTM and no averaging layers) in order to avoid an overfitting of the data. At the end of the first cycle, the quality of the prediction was good enough to allow the discrimination of substituted from unsubstituted reads for the 40 to 100% BrdU samples. Reads were relabelled as substituted or unsubstituted based on these results, and a second training cycle was run. This allowed the separation of substituted from unsubstituted reads for the 20 and 30% BrdU samples. The final network (RepNano v2 architecture) was trained on this cleaner dataset of 400 reads per sample.

#### Megalodon model training

We performed a first training of our model with ONT’s Taiyaki software on 400 reads from each of the 11 BrdU samples described above basecalled with RepNano v2 using a LSTM architecture referred to as mLstm_cat_mod_flipflop with default parameters. Meanwhile, we selected 100 reads from the 0% BrdU sample where forks had been detected, corresponding to false positives (these were mainly found in the rDNA and at the positions of Ty elements). We also selected 100 reads in the same regions from the 100% BrdU sample not to introduce a bias in the neural network at these locations. These reads were added to the outputs of the first model to perform a second training. Finally, the Taiyaki model was converted into a Guppy model.

### BrdU basecalling and read mapping

Basecalling and read mapping were performed from MinKNOW-generated fast5 files upon sequencing using Megalodon, that combines Guppy basecalling (using the model trained as described above to detect BrdU at the nucleotide level) and read mapping to the reference genome (see basecalling_sample.sh script on NanoForkSpeed GitHub repository). This step was carried out using a GPU-enhanced computer to allow fast processing. As the resulting BAM files could be quite big, a custom R^58^ script (R version 4.0.3) named basecalling_sample.r first split BAM files into 50,000 read subfiles. The parsing was then performed in R with the mega_parsing homemade function. For each read, this function imported from the BAM file the read identity (read_id), the DNA strand, the chromosome name, the start position and length of the read, the sequence and the Mm and Ml fields which contain the position and output score of detected BrdUs, respectively, using Samtools (version 1.10). The function then allocated a probability of being a BrdU at every position corresponding to a thymidine in the mapped sequence. All discontinuities (gaps) longer than 100 nucleotides introduced during the mapping step were recorded; this information was used after the fork detection procedure to filter out forks overlapping such gaps as discontinuities in the BrdU signal may interfere with fork speed measurement. Data were then smoothed using a combination of a rolling mean on 100 nucleotides and a Gaussian weighted rolling mean on 2.5 kb, with exclusion of the first and last 2.5 kb windows (which removed reads <5 kb from our analysis). This smoothing procedure was designed to allow the piecewise linear simplification (see below) without altering the shape of the signal too much. The output file contained both the raw and the smoothed signal as well as the mapping information (read_id, chromosome name, start and end positions, strand and gap positions).

### BrdU basecaller comparison

For each of the 11 genomic DNA samples with different BrdU substitution rates described above, a set of 8000 nanopore reads were basecalled either with Megalodon or with DNAscent v1^15^, DNAscent v2^16^, RepNano^13^ transition matrices (RepNano_TM) and RepNano convolutional neural network (RepNano_CNN). RepNano_TM and RepNano_CNN were used as in ref. ^13^. For each basecaller, the BrdU content was computed in 1 kb bins for every read. The overall BrdU content of a given DNA sample corresponded to the mean of the BrdU content values per 1 kb window. Means of the 11 samples were plotted against BrdU contents measured by mass spectrometry (MS) in Supplementary Fig. 1a. The proportionality between basecaller and MS estimates was assessed using a linear regression and by computing the mean square error between the observed and ideal lines. Distributions of BrdU content values per 1 kb window for the reads from the 11 samples were represented as normalized densities using the geom_freqpoly() function of the ggplot2 R package in Supplementary Fig. 1b–f. Although reads used for the analyses shown in Supplementary Fig. 1 came from a different sequencing run than reads used for the training of Megalodon, we cannot completely exclude that Megalodon’s performance might benefit from having been trained on DNA originating from the same samples as those used to compare basecallers.

### Fork detection and orientation

After pulse-labelling, BrdU signals corresponding to ongoing replication forks are visualized on nanopore reads as a steep ascending slope starting from a segment of null BrdU content followed by a shallower decreasing slope; the steep and shallow slopes reflect BrdU incorporation during the pulse and the chase, respectively, and this signal asymmetry allows the determination of the direction of fork progression^13^. Fork detection and orientation were performed with custom R scripts using a piecewise linear simplification approach deriving from the original FORK-seq manuscript^13^. Reads were first converted into a series of segments using the Ramer-Douglas-Peucker algorithm^59,60^ (Hausdorff distance epsilon = 0.1, using DouglasPeuckerEpsilon function of the kmlShape R package). Only reads with 3 or more segments were kept as reads with less segments could not form a complete fork. Segments were classified into 4 categories using their slope and mean BrdU signal (B = flat segment with a background BrdU level, A = flat segment with a BrdU level above background, P = segment with a positive slope and N = segment with a negative slope). In order to set the background threshold, the distribution of the mean BrdU signal on 1 kb windows computed for the reads containing at least 3 segments of a given experiment was plotted; in every experiment, DNA replicated before the BrdU pulse (i.e., DNA with a theoretical null BrdU signal) was separated from the rest of the DNA by local minimum near 0.02, which was therefore set as the background threshold (b2a.thr parameter) for all samples.

We were specifically interested in capturing the segment corresponding to BrdU incorporation during the pulse, that is the section between the starting and ending points of the steep slope, in order to extract fork speed defined as the length of DNA replicated during the duration of the pulse (i.e., 2 min) divided by the labelling time. We thus focused on pulse segments for which we could determine the start and end positions. We determined two patterns to identify forks depending on their orientation on the nanopore reads. Rightward forks must be preceded by a B segment, then must contain one or several P segments that may be interrupted by A segments owing to noise and then at least one N segment; they must not be directly followed by a B segment as the BrdU level corresponding to the thymidine chase is above background. We then used a regular expression approach to recognize the “BP(P|A)*N+” pattern, excluding forks for which the following segment was a B. Leftward forks were identified thanks to a symmetrical pattern “P+(N|A)*NB”, excluding forks for which the preceding segment was a B. This prevented any overlap between forks detected on a given read (forks must start with a B segment and cannot end on a B segment) and excluded incomplete replication tracks as well as symmetrical signals due to pairs of forks initiated after the start of the pulse or terminated before the end of the pulse, for which the actual labelling time could not be precisely estimated. Furthermore, in case of multiple replication signals on a single read detected as a succession of leftward and rightward forks, we could determine the position of individual initiation (termination) sites defined as the midpoints between diverging (converging) forks.

Our procedure was performed using the homemade NFSmaster function of the NFS_function.r script. It creates an output file saved in the .rds format in which the data are organised as a list of 4 elements: (1) a list of tibble (specialised type of data.frame in the R tidyverse) containing (1.1) the reads filtered according to their length and the presence of 3 or more linearized segments and (1.2) the reads with detected forks; (2) a tibble of all the detected forks; (3) a tibble containing the detected initiation (Ini) and termination (Ter) events; (4) a table summary of different metrics of the experiment. Fork detection was performed on the split data corresponding to the basecalling of 50,000 reads and results were merged using the NFS_merging function of the NFS_function.r file. Merged files have the same organization with a slightly simplified summary table but do not contain reads without fork; they were used to produce the figures and data discussed in this manuscript.

### Fork speed analysis

Fork data from every read and experiment were collected in a master data table containing read information (read_id, chromosome name, read start and end positions, read strand), fork parameters (X0 = start of BrdU incorporation = position of the B/P and N/B transitions for rightward and leftward forks, respectively; X1 = start of the thymidine chase = position of the (P|A)/N and P/(A|N) transitions for rightward and leftward forks, respectively; speed in bp/min = fork speed averaged over the 2 min of the pulse = absolute value of (X1 − X0)/2; direction = Left or Right; type = leading or lagging; d.Y = BrdU signal amplitude at X1 position minus BrdU signal amplitude at X0 position) and experimental parameters (Exp = name of the experiment; B_pulse = BrdU concentration in the medium during the pulse (in µM); t_pulse = duration of the pulse (2 min in all experiments); T_chase = thymidine concentration in the medium during the chase (in µM); temp = growing temperature (in °C); strain = yeast strain used; mutant = wild-type (WT) or name of the inactivated gene; HU = hydroxyurea concentration in the medium (in mM)). Data were filtered using these parameters to group fork speeds according to the criteria presented in the figures of this manuscript. Detailed information for every sample sequenced in our study are presented in Supplementary Data 1.

Computing fork velocity from reads of increasing minimal length resulted in a negligible rise in the median fork speed estimated by NFS, confirming that a 2 min BrdU pulse duration was adequately suited with respect to read length to detect short as well as long tracks in our experiments; the median length of reads with forks is indicated for each sample in Supplementary Data 1.

### Genomic map of fork speed

Forks detected in all BT1 biological replicates grown in standard conditions at 30 °C were converted into GenomicRanges using the GenomicRanges R package and reduced to their centre. The velocities of these forks were then binned into non-overlapping windows of different width (1, 2, 3, 4, 5, 6, 7, 8, 9, 10, 15 and 20 kb). The number of forks (coverage), the median speed and the 98% confidence interval of the median (using the MedianCI function of the DescTools R package) were computed for every window. In addition, a Mann–Whitney–Wilcoxon test was performed to compare the speed distribution in a given window to the speed distribution on the whole genome. *P*-values for slower and faster speed were corrected for the multiplicity of testing using the Holm method from the p.adjust function, taking into account the number of windows for all scales. Results were saved as bigwig files and used for plots in Fig. 5 and Supplementary Figs. 8, 9 and 11, setting statistical significance to *p* < 0.01. Bins with no fork were tagged N/A. In order to distinguish meaningful changes in local fork velocity distribution, the experimental map was superimposed to a randomized speed map. To generate this map, fork locations were kept unchanged but speed values were randomized 1000 times and binned into 20 kb (Fig. 5 and Supplementary Fig. 8) or 1 kb (Supplementary Figs. 9 and 11) windows. For each bin and each randomization, the median speed was computed, generating 1000 medians of randomized speeds per bin. The overall median and the 1st and 99th percentiles were extracted from these data for every bin and plotted as the median speed and 98% confidence interval of the median to build the shuffled speed map. In Fig. 5 and Supplementary Figs. 8 and 11, genomic feature coordinates are from the UCSC SGD_other track and replication origins (ORIs) are from ref. ^21^. Oriented genes in Supplementary Fig. 9 are from the Saccharomyces_cerevisiae.R64-1-1.104.gtf file from Ensembl (see “Computational resources” below for further details).

### Replication fork directionality (RFD) computation

RFD is calculated for a given position as the difference between the proportions of rightward- (R) and leftward- (L) moving forks (RFD = R − L). RFD profiles from MCM869 FORK-seq and OK-seq data were computed as in ref. ^13^. BT1 RFD profile from NFS data (i.e., X0 and X1 coordinates of oriented forks) was generated in a similar way with the simpleRFD function of the script helper_function.r (this function produces 4 bigwig files corresponding to the RFD and the total, leftward and rightward forks coverage) using forks from all BT1 biological replicates grown in standard conditions at 30 °C. RFD data were binned into non-overlapping 100 nucleotide windows to reduce the size of the plot files. The correlation table in Supplementary Fig. 3b reporting Spearman’s pairwise correlation coefficients was produced using the cor.rfd function of the helper_function.r script and the ggcorrplot function of the ggcorrplot R package. The cor.rfd function rests on the base R cor function but works on a coverage type of data (RleList) and excludes positions where N/A are present in at least one of the RFD profiles for which the correlation is computed.

### Analysis of initiation and termination events

Initiation (ini) and termination (ter) event positions, defined as the midpoints between diverging and converging forks, respectively, were extracted from all BT1 biological replicates grown in standard conditions at 30 °C and processed as in ref. ^13^. Replication origins (ORIs) are from ref. ^21^. Distance of each ini or ter to the centre of the nearest ORI was computed using the distanceToNearest function of the GenomicRanges R package. Empirical cumulative distribution functions were plotted using the Ecdf function of the Hmisc R package. Shuffled versions of ini and ter positions were produced as a control using the custom shuffleGRen function of the helper_function.r script, which randomizes GenomicRanges positions while conserving the number of events *per* chromosome.

### Simulation of BrdU incorporation during DNA replication

We simulated reads containing a BrdU signal mimicking an elongating fork as well as reads bearing multiple forks. Simulations were performed using Python scripts (Python version 3.6).

#### Simulation of BrdU level

We modelled intracellular BrdU level *b(t)* as a function of time *t* as (i) zero before the BrdU pulse; (ii) an ascending section modelled by an exponential increase ${As}(t)={M}(1-{\exp }(-\frac{t}{{\tau }_{1}}))$ where $t$ is the time since the start of the pulse, *M* the saturating concentration of BrdU and ${\tau }_{1}$ the characteristic time of BrdU intake; (iii) a decreasing section starting at the start of the chase at time ${t}_{{{{{{{\rm{pulse}}}}}}}}$ (also corresponding to the duration of the pulse) that was modelled by a similar exponential decrease from the value attained at $t={t}_{{{{{{{\rm{pulse}}}}}}}}$: ${Ds}(t)={As}({t}_{{{{{{{\rm{pulse}}}}}}}})+(m-{As}({t}_{{{{{{{\rm{pulse}}}}}}}}))(1-{\exp }{{\mbox{(-}}}\frac{t-{t}_{{{{{{{\rm{pulse}}}}}}}}}{{\tau }_{2}}{{\mbox{)}}})$, where *m* is the asymptotical BrdU concentration and ${\tau }_{2}$ the characteristic time of BrdU outtake.

#### Simulation of a single replication fork

Given a fork speed *v* and a start position used as *x* = 0, BrdU time pulse shape was converted into a spatial BrdU incorporation pattern *BrdU(x)* using the simple relation: *BrdU(x)* *=* *b(x/v)*. 10,000 reads containing a single fork were simulated with or without noise (see below) for the analyses presented in Fig. 3a and Supplementary Fig. 7.

#### Simulation of multiple replication forks

To simulate multiple forks, we created a DNA segment of 300 kb in length. We then randomly positioned 6 origins on it to have on average an origin every 50 kb. The firing time of each origin was randomly chosen between 0 and 30 min. A fork speed randomly drawn from a given distribution (see below) was assigned to the whole segment, which was simulated only if firing times, origin positions and fork speed created a set of 12 forks. Next, the moment of the pulse was randomly chosen between the firing time of the earliest origin and the three-fifths of the firing time of the latest origin (this time interval was chosen because it allowed to obtain replication signals in most simulated reads). Finally, to simulate molecules of similar length as experimental reads, the DNA segment was sliced into fragments according to a truncated log-normal distribution (truncations at 5 and 300 kb) with a shape parameter of 0.5 and a scale of 350. 100,000 reads containing one or several forks were simulated with or without noise (see below) for the analyses in Fig. 3a and Supplementary Fig. 7.

#### Choice of the simulation parameters

A replication fork was modelled by two shifted exponentials, each having two parameters ($M$, $\,{\tau }_{1}$ and $m$, $\,{\tau }_{2}$ for the ascending and descending parts, respectively; see above). To simulate replication signals resembling those found on real sequencing reads, we used parameters coming from experimental data. To do so, we selected among the >125,000 individual forks detected by NFS in BT1 cells grown at 30 °C those for which the chase was long enough (>2.5 kb), fitted the ≈90,000 forks meeting this criterion using the model described in the ‘Simulation of BrdU level’ section and the speed measured by NFS, and extracted fork parameters. In order to retain potential correlations between parameters, all four parameters of a randomly chosen experimental fork were assigned to a simulated fork. Fork speed *v* was randomly chosen from the deconvolved true fork speed distribution (see below) except when indicated otherwise.

#### Adding simulated experimental noise to the signal

To take into account signal variability between reads, we added to the *BrdU(x)* signal a read-dependent offset value (*O*) drawn from a log-normal distribution of shape 1.98, location −4.09e−06 and scale 0.001 fitting the BrdU signal distribution of nanopore reads of DNA with no BrdU labelling. *O* was drawn again if its value was >0.2 or <1e−7. *BrdU(x)+O* signals reaching values >1 were truncated to 1. We then randomly assigned “Nan” (Not a number) values to ≈77% of the signal so that only thymidine (T) positions had information about the BrdU content. Finally, to mimick Megalodon’s output signal, which is a probability of being a BrdU at each T site peaking at either 0 or 1, we assigned to each T position a value *B* drawn from a binomial law B*(n,p)* with parameters *p* = *BrdU(x)*+*O* and *n* randomly chosen in [1,2,3]; *B* was subsequently divided by *n* to normalize between 0 and 1. Visual inspection of simulated versus experimental reads confirmed that they were virtually indistinguishable.

### Mean BrdU traces and signal noise analysis for experimental and simulated forks

The experimental and simulated mean BrdU traces of oriented replication signals in Supplementary Fig. 6f, g were computed from ≈90,000 forks selected among the >125,000 detected by NFS in BT1 cells grown at 30 °C for which the chase was >2.5 kb (please note that leftward forks were reoriented in the rightward direction) and from 10,000 simulated reads containing a single fork, respectively, with the BrdU signal being smoothed using a 100 bp running average to have information even at non-thymidine sites. In order to characterize signal fluctuations (i.e., signal noise) in experimental and simulated data, the autocorrelation of the mean BrdU signal smoothed at 100 bp minus the signal smoothed at 1000 bp was computed. Subtracting the signal smoothed at 1000 bp removed slow BrdU variations from each track to solely keep local fluctuations that define signal noise.

### Analysis of simulated data

The smoothing process of simulated reads during the parsing procedure and fork detection with NFS was performed with the same settings as for experimental reads. Speed error was computed by subtracting, for each simulated replication fork, its true speed to the speed estimated by NFS. For simulated reads with multiple replication forks, all forks within a given read had the same speed. To estimate NFS error for different speed categories, true speeds from simulated reads were grouped into bins of 100 bp/min.

### Estimation of the true fork speed distribution in yeast from experimental measurements

A measured fork speed is an approximation of the true fork speed as (i) BrdU incorporation and detection by nanopore sequencing are subject to noise, and (ii) the measurement by NFS is sensitive to the noise and amplitude of BrdU signals in a complex manner. We used simulated reads to estimate the “transfer function” between the true and the observed fork speed distributions allowing us to deconvolve the measurement errors from the observed fork speed distribution and in turn to estimate the true speed distribution underlying the observed speed distribution. We first built a library of 100,000 reads with known true speeds following a uniform distribution between 50 and 5000 bp/min. We processed each noisy BrdU incorporation profile with NFS and obtained, for each true speed, the response fork speed distribution of the experimental procedure. We then determined the weight of each true speed category required to recover the experimentally observed distribution of >125,000 fork velocities at 30 °C. The weights corresponded to the deconvolved true fork speed distribution. The computational details of this procedure are detailed in the following paragraph.

We considered 44 discretised true fork speed values *v*_*k*_ = k*100 bp/min for 1 ≤ *k* ≤ 44. The 100,000 simulated reads following the uniform speed distribution were then affected to 44 classes if their true speed fell inside a window centred on *v*_*k*_ and of width 400 bp/min, i.e., reads belonged to more than one class. This strategy was adopted in order to have enough reads in each class. Then, for each class *k*, we collected fork speeds estimated by NFS processing, leading to NFS fork speed distribution in response to true speed *v*_*k*_ that was subsequently fitted with a five-component Gaussian mixture. We then determined the weight *w*_*k*_ so that the weighted sum of those 44 distributions best fitted the experimental fork speed distribution. The weights *w*_*k*_ are an estimate of the true fork speed distribution within the 44 classes. This procedure was repeated independently 5 times and the average of the 5 weight sets were used as the deconvolved true fork speed distribution. Finally, this discretised distribution was fitted using a Gaussian mixture with six components to create a continuous true fork speed distribution. To evaluate the deconvolution procedure, we generated a new set of 100,000 reads with multiple forks following the estimated true fork speed distribution and processed them with NFS. The obtained observed fork speed distribution closely resembled the experimental distribution of >125,000 fork velocities, validating our approach. Deconvolution was performed with custom Python scripts using a library from ref. ^61^.

The mode of the estimated true speed distribution in Fig. 3b was determined using the mlv1 function of the modeest R package. The major peak of the distribution was fitted with a normal distribution with a mean equal to this mode. Standard deviation and weight of the normal distribution were then adjusted to best fit the peak.

### Fork speed versus replication timing

Replication timing (RT) data are from ref. ^42^. The liftOver tool (http://hgdownload.soe.ucsc.edu/goldenPath/sacCer1/liftOver/) was used to convert genomic positions from sacCer1 to the sacCer3 version of the yeast genome. Data were normalized between 0 and 1 (start and end of S phase, respectively). The mean RT was computed for each fork, then fork speed versus RT was plotted as a 2D density plot with hexagonal bins using the geom_hex() function of the ggplot2 R package with default parameters. Spearman’s correlation between fork speed and RT was computed using the stat_cor() function of the ggpubr R package.

### Statistical analysis

The R environment v4.0.5 was used for all the analyses. Prior to analysis, fork speed values were averaged for every sample according to the classes of the studied factors, in order to (1) decrease the sensitivity of the tests, which otherwise detect fork speed differences of no practical significance when extremely large number of values are compared and (2) use as a unique source of variation the inter-experiment variations, which correspond to error variations, while fork speed variations have a biological origin. Data were fitted to a linear model that included fork speed as response variable, the factor of interest as predictive variable and the biological replicate/sequencing run as blocking factor. Two by two effect comparisons (two-sided contrast comparisons) were performed with the emmeans() function of the emmeans R package. When only two values were present in one group (Fig. 4a, b and Supplementary Fig. 12), a limma linear fitting and contrast analysis was performed with the limma R package. Statistical significance was set to *p* ≤ 0.05. In each case, type I error was controlled by correcting the *p*-values according to the Benjamini & Hochberg method (“BH” option in the p.adjust() function of R). Speeds of forks overlapping two different features (e.g., a tRNA and a centromere) or two tRNAs were removed from the analyses presented in Fig. 6a, b and Supplementary Fig. 12 to respect the exclusive factor level requirement of statistical analysis. For the examination of speed at individual tRNAs (Supplementary Fig. 12), means above 5000 bp/min or showing a leverage of 1 in the fitted model were removed from the analysis as they strongly influenced the fitting. This concerned 11 values among the 2984 tRNA x sequencing run levels analysed. All samples corresponded to independent cell cultures except for the two BT1 PromethION runs, which were technical replicates (two sequencing libraries from the genomic DNA of one BT1 cell culture analysed on different PromethION flow cells); the “library plus flow cell” effect was deemed sufficient to consider these as distinct samples. Statistical analysis results are detailed in Supplementary Table 2.

### Visualisation tools

Plots were made using custom R scripts. For half-eye plots, we used the stat_slab and stat_pointinterval functions of the ggdist R package. stat_slab outputs a density distribution of the experimental points; stat_pointinterval outputs the median and the 50% and 95% intervals of the data centred on the median.

### Reference genome

The sacCer3 genome release (S288C_reference_sequence_R64-2-1) was used as the reference genome. The rDNA locus is composed of two ribosomal DNA units on chromosome XII (chrXII: 451,575–468,931) in this version. As rDNA reads containing more than two repeats tended to incorrectly map and to create artefactual BrdU signals, we added an artificial chromosome with 10 tandem rDNA repeats to the reference genome. The external rDNA repeats were flanked by 10 kb sequences located upstream and downstream of the original rDNA locus to allow the mapping of reads overlapping the first or last rDNA repeat of the rDNA array. In order to build this extra “chromosome”, 8 copies of the second rDNA unit (chrXII: 459,793–468,929 fragment) were inserted between the first and second unit of the original S288C genome, resulting in a 113-kb-long “chromosome” that we named rDNA-10R. Because the proper alignment of reads on tandem repeats the unit of which is of similar length to that of the reads requires specialised procedures, reads mapping to rDNA-10R, which accounted for the most part of rDNA reads, were subsequently excluded from our analyses.

### Computational resources

Genomic feature coordinates (centromeres, telomeres, HML/HMR loci, rDNA and tRNA genes) were extracted from the UCSC SGD_other track (https://genome.ucsc.edu/cgi-bin/hgTables) and saved as bed files (script Script_YeastAnnotation.r). Please note that “telomere” annotations in the SGD_other track encompass both the terminal stretch of telomeric repeats (when present in the sacCer3 genome) and subtelomeric sequences; moreover, since our smoothing procedure removes the first and last 2.5 kb of each read, telomeric repeats were excluded from our analyses. The UCSC SGD_other track was also used to determine the direction of transcription of tRNA genes in Fig. 6b and in Supplementary Fig. 12. In this figure, tRNA genes were subsequently categorized as co-directional (CD) or head-on (HO) with respect to replication if their transcription was in the same or opposite orientation to the main direction of fork progression, respectively, which was determined for each tRNA according to its mean RFD value (RFD > 0, forks travel mostly rightward; RFD < 0, forks travel mostly leftward). In Fig. 6a, b and Supplementary Figs. 10 and 12, forks were classified according to their overlap with either a group of features (centromeres, telomeres and tRNA genes) or individual features (rDNA locus and individual centromeres, telomeres and CD/HO-sorted tRNA genes) after fork conversion into GenomicRanges. The overlap was tested with the OverlapsAny function of the GenomicRanges R package (minimal overlap was one nucleotide). Yeast replication origins in Fig. 5 and Supplementary Figs. 3a, c, 8 and 11 are from ref. ^21^. RT data in Fig. 6d are from ref. ^42^ (data accessible from NCBI’s Gene Expression Omnibus repository, accession code GSM1036187, GSM1036187_T7107_normalised.wig.gz file). Oriented genes in Supplementary Fig. 9 are from the Saccharomyces_cerevisiae.R64-1-1.104.gtf file downloaded from Ensembl (http://ftp.ensembl.org/pub/release-104/gtf/saccharomyces_cerevisiae/). DNAscent v1 and v2 were downloaded from https://github.com/MBoemo/DNAscent.

### R packages

R packages used in this study are kmlShape version 0.9.5 (https://CRAN.R-project.org/package=kmlShape); DescTools version 0.99.44 (https://CRAN.R-project.org/web/packages/DescTools); RcppRoll version 0.3.0 (https://CRAN.R-project.org/package=RcppRoll); Hmisc version 4.6-0 (https://CRAN.R-project.org/package=Hmisc); tidyverse^62^; GenomicRanges^63^; rtracklayer^64^; BSgenome version 1.56.0 (https://bioconductor.org/packages/BSgenome); ggdist version 3.0.1 (https://mjskay.github.io/ggdist/); patchwork version 1.1.1 (https://CRAN.R-project.org/package=patchwork); ggplot2 version 3.3.5 (https://CRAN.R-project.org/package=ggplot2); ggcorrplot version 0.1.3 (https://CRAN.R-project.org/package=ggcorrplot); ggpubr version 0.4.0 (https://CRAN.R-project.org/package=ggpubr); gridExtra version 2.3 (https://CRAN.R-project.org/package=gridExtra); modeest version 2.4.0 (https://CRAN.R-project.org/package=modeest); ggprism version 1.0.3 (https://CRAN.R-project.org/package=ggprism); ggrepel version 0.9.1 (https://CRAN.R-project.org/package=ggrepel); furrr version 0.2.3 (https://CRAN.R-project.org/package=furrr); devtools version 2.4.2 (https://CRAN.R-project.org/package=devtools); emmeans version 1.5.5-1 (https://CRAN.R-project.org/package=emmeans); and limma version 3.46.0 (https://bioconductor.org/packages/release/bioc/html/limma.html).

### Genomic coordinates

Coordinates are given according to the sacCer3 yeast genome assembly.

### Reporting summary

Further information on research design is available in the Nature Research Reporting Summary linked to this article.

## Supplementary information

Supplementary Information

Description of Additional Supplementary Files

Supplementary Data 1

Reporting Summary

## Data Availability

Nanopore sequencing data generated in this study have been deposited in the ENA database under accession code PRJEB50302. Source data are available at https://github.com/LacroixLaurent/NanoForkSpeed and 10.5281/zenodo.5958270. Yeast genomic feature coordinates used in this study originate from UCSC SGD_other track (https://genome.ucsc.edu/cgi-bin/hgTables), oriented genes are from Ensembl database (Saccharomyces_cerevisiae.R64-1-1.104.gtf file available at http://ftp.ensembl.org/pub/release-104/gtf/saccharomyces_cerevisiae/), replication origins are from ref. ^21^ and replication timing data are from ref. ^42^ (data accessible at NCBI GEO database, accession code GSM1036187). Source data are provided with this paper.

## Source data

Source Data

## Code availability

Megalodon-based BrdU basecaller, NFS software and associated R scripts can be accessed at https://github.com/LacroixLaurent/NanoForkSpeed and 10.5281/zenodo.6535390. Custom R scripts for statistical analyses can be accessed at https://gitlab.pasteur.fr/gmillot/anova_contrasts/-/tree/v7.2.0. Python scripts are available at https://github.com/organic-chemistry/simunano.
