# Supplementary material for: Genome-wide mapping of individual replication fork velocities using nanopore sequencing
Source: Nat Commun. 2022 Jun 8;13:3295. doi: 10.1038/s41467-022-31012-0 (PMC9177527; doi:10.1038/s41467-022-31012-0)
Supplement: Supplementary file 1 — Supplementary Information [file 41467_2022_31012_MOESM1_ESM.pdf]

Genome-wide mapping of individual replication fork velocities using nanopore sequencing

SUPPLEMENTARY INFORMATION

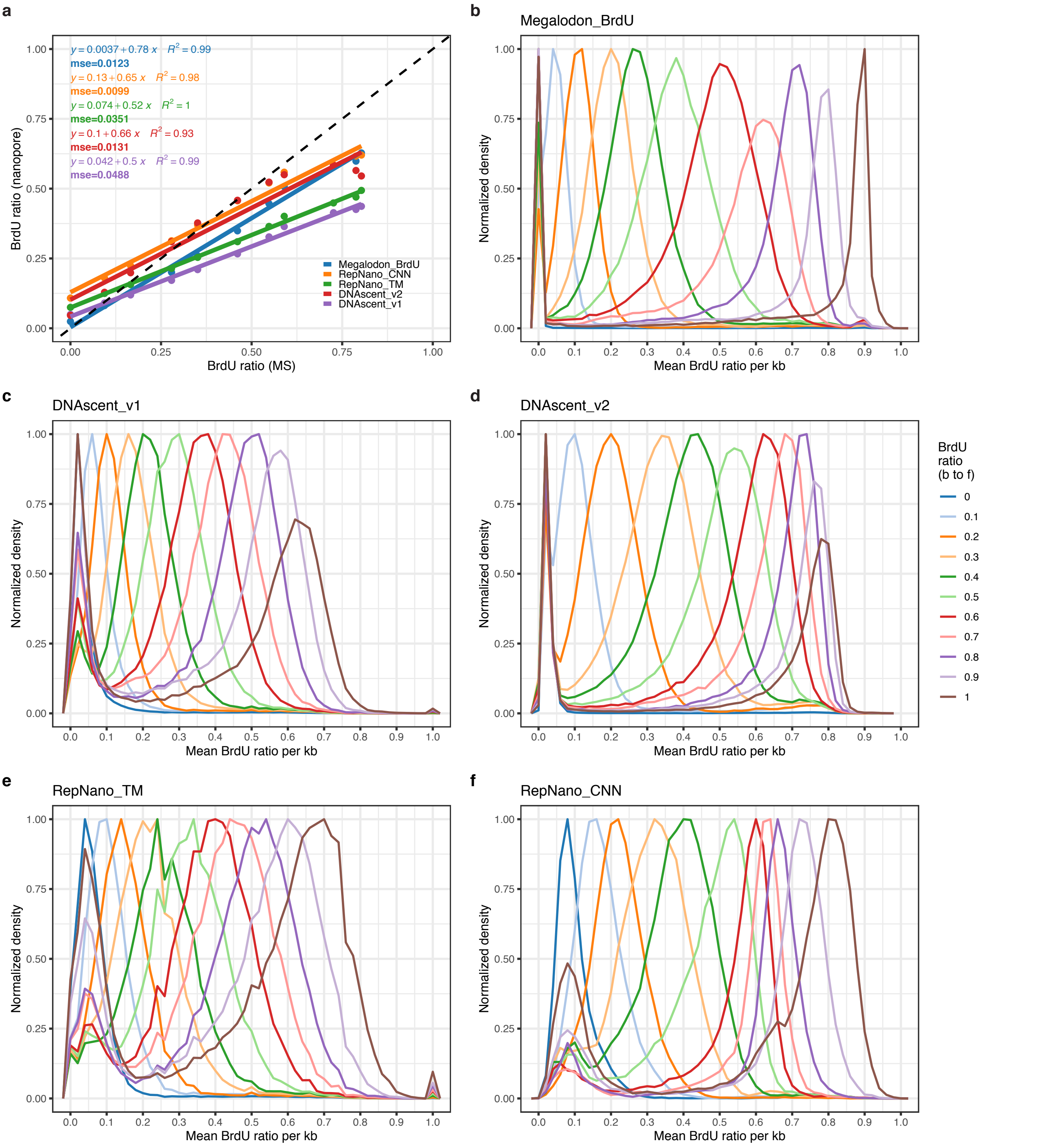

Supplementary Figure 1. Detection of BrdU in genomic DNA by Megalodon. a, Overall BrdU content estimated using Megalodon, DNAscent v1[15], DNAscent v2[16], RepNano[13] transition matrices (RepNano_TM) and RepNano convolutional neural network (RepNano_CNN) *versus* mass spectrometry measurements (MS) for 11 samples of genomic DNA from MCM869 cells grown with a percentage of 0 (thymidine control) to 100% BrdU in the culture medium with 10% increments. R^2, regression line R-squared value; mse, mean squared error relative to the perfect correlation line (y = x, dashed line). Regression line equations are indicated to compare slopes and intercepts; the equation for Megalodon is the closest to y = x. **b-f**, Normalized density of BrdU content averaged over 1 kb for nanopore reads of the 11 samples with different proportions of BrdU in the culture medium (0 to 1 with 0.1 increments) described above as determined using Megalodon (**b**), DNAscent (**c**), DNAscent v2 (**d**), RepNano transition matrices (**e**) and RepNano convolutional neural network (**f**) detection methods.

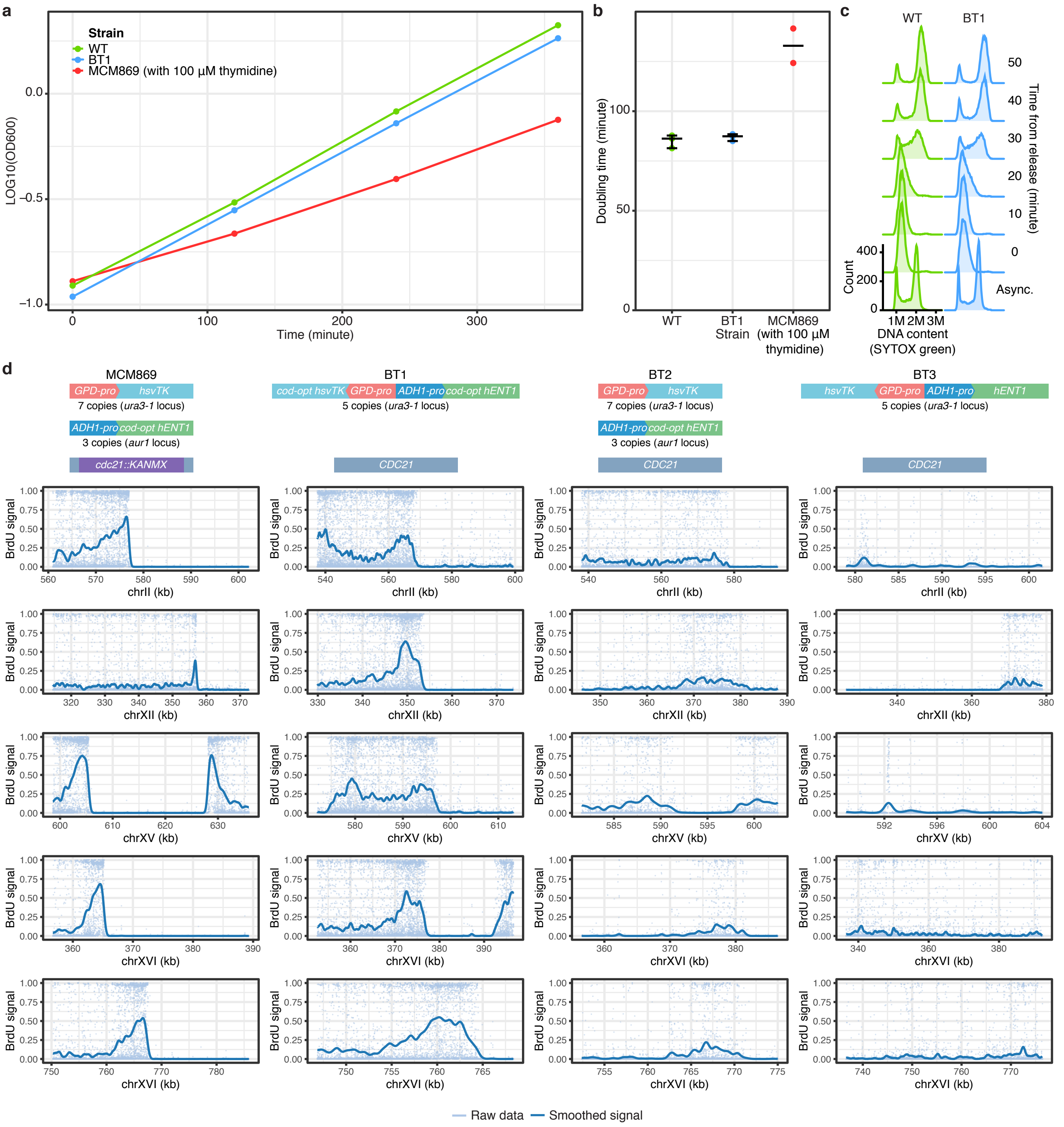

Supplementary Figure 2. Characterization of the BT1 yeast strain. a-c, BT1 cells exhibit WT growth and S phase progression. **a**, Representative growth curve of WT, BT1 and MCM869 cells with 100 µM thymidine. This experiment was performed independently three times for WT and BT1 cells, twice for the MCM869 strain, with similar results. **b**, Doubling time of WT, BT1 and MCM869 cells with 100 µM thymidine (black centre line and whiskers, median and extreme values, respectively; n values are independent cell cultures: $n_{WT} = 3$, $n_{BT1} = 3$ and $n_{MCM869} = 2$). **c**, Representative FACS analysis of S phase progression of BT1 cells. WT and BT1 cells were arrested in G1 with α-factor and released synchronously into S phase. DNA content was analysed by flow cytometry after DNA staining with SYTOX Green. This experiment was performed twice independently with similar results. Async., asynchronous. **d**, Examples of BrdU content profiles of nanopore reads with BrdU signal above background level mapping to the same genomic regions from pulse-labelled MCM869, BT1, BT2 (MCM869 strain with a functional *de novo* dTMP biosynthesis pathway) and BT3 (strain expressing hsvTK and hENT1 from five tandemly integrated copies of the p306-BrdU-Inc plasmid[17]) cells using Megalodon. A scheme below the name of each strain recapitulates the integrated transgenes allowing the incorporation of exogenous thymidine or thymidine analogues in the cognate strain, and specifies if endogenous thymidine synthesis was disrupted through inactivation of the thymidylate synthase-encoding *CDC21* gene. The number of integrated copies is indicated for each transgene; the name of the locus of insertion is given in parentheses. Diagrams are not drawn to scale. Pro, promoter; cod-opt, codon-optimised for expression in yeast. Light blue dots, raw data from Megalodon (dots represent the probability of BrdU at each thymidine position); blue curve, smoothed signal.

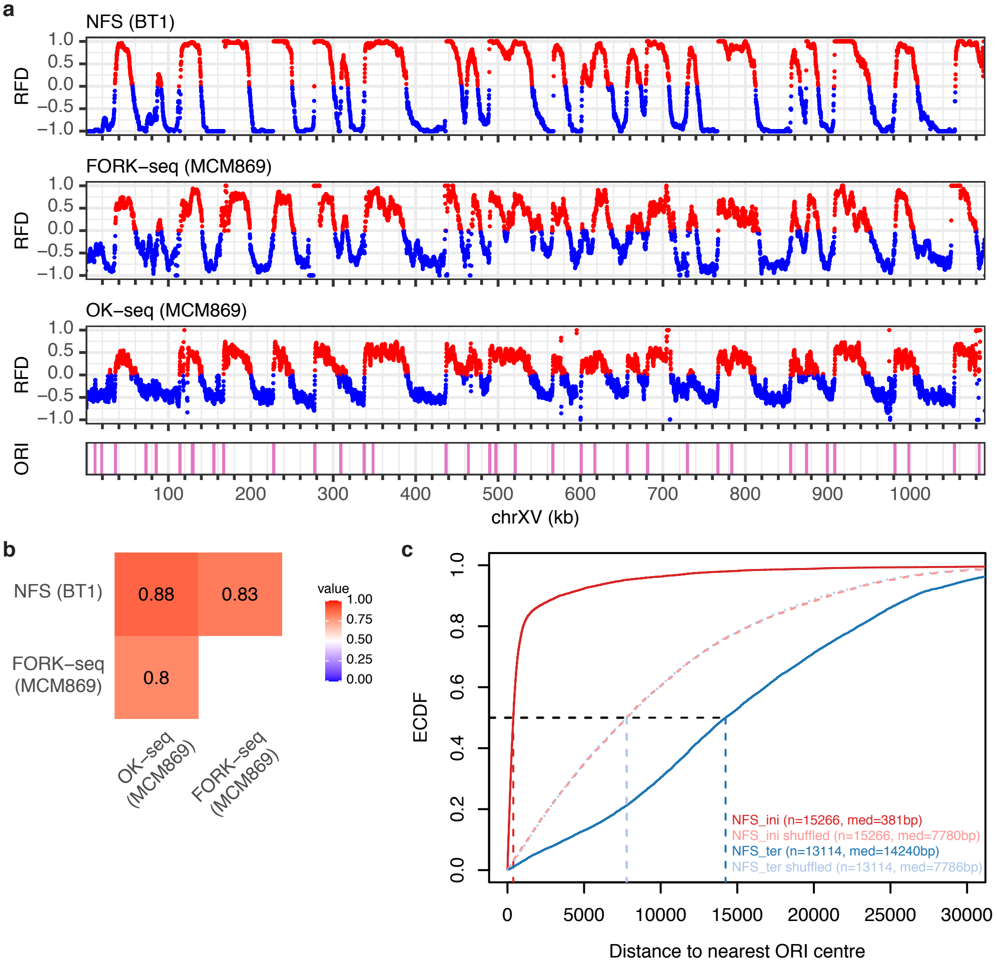

Supplementary Figure 3. RFD profiling and detection of initiation and termination events using NFS. a, RFD profiles in 100 bp adjacent windows of chromosome XV from NFS, FORK-seq and Okazaki fragment sequencing (OK-seq)[13]. Bottom, known *S. cerevisiae* replication origins (ORI) from[19]. **b**, Spearman's pairwise correlation coefficients between genome-wide RFD profiles obtained by the indicated methods. **a**, **b**, The strain name is given in parentheses. **c**, Empirical cumulative distribution function (ECDF) of the distances between diverging (NFS_ini, red) or converging (NFS_ter, blue) fork midpoints and the closest ORI centre[19]. Shuffled versions of NFS_ini (pink) and NFS_ter (light blue) positions were used as a control. ini, initiation event; ter, termination event; n, number of ini or ter; med, median distance between ini or ter and ORI centres.

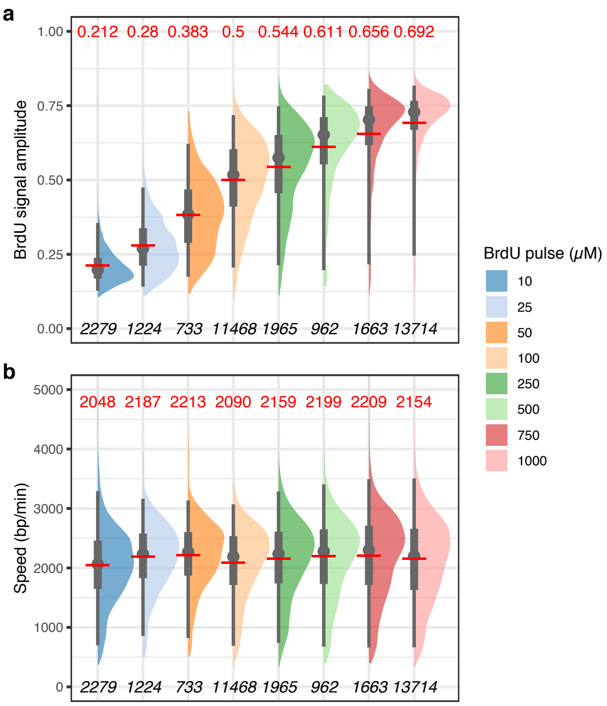

Supplementary Figure 4. Impact of BrdU concentration used for the pulse-labelling of replicating DNA on fork speed measurement by NFS. a, **b**, Half-eye plots of fork maximal BrdU signal amplitudes (**a**) and of fork velocities (**b**) determined by NFS on nanopore reads of genomic DNA from cells pulse-labelled with BrdU doses ranging from 10 µM to 1 mM. Red line, mean amplitude or speed, value indicated in red on top; grey dot, median; thick and thin grey vertical lines, 50 and 95% intervals, respectively; bottom, number of measurements.

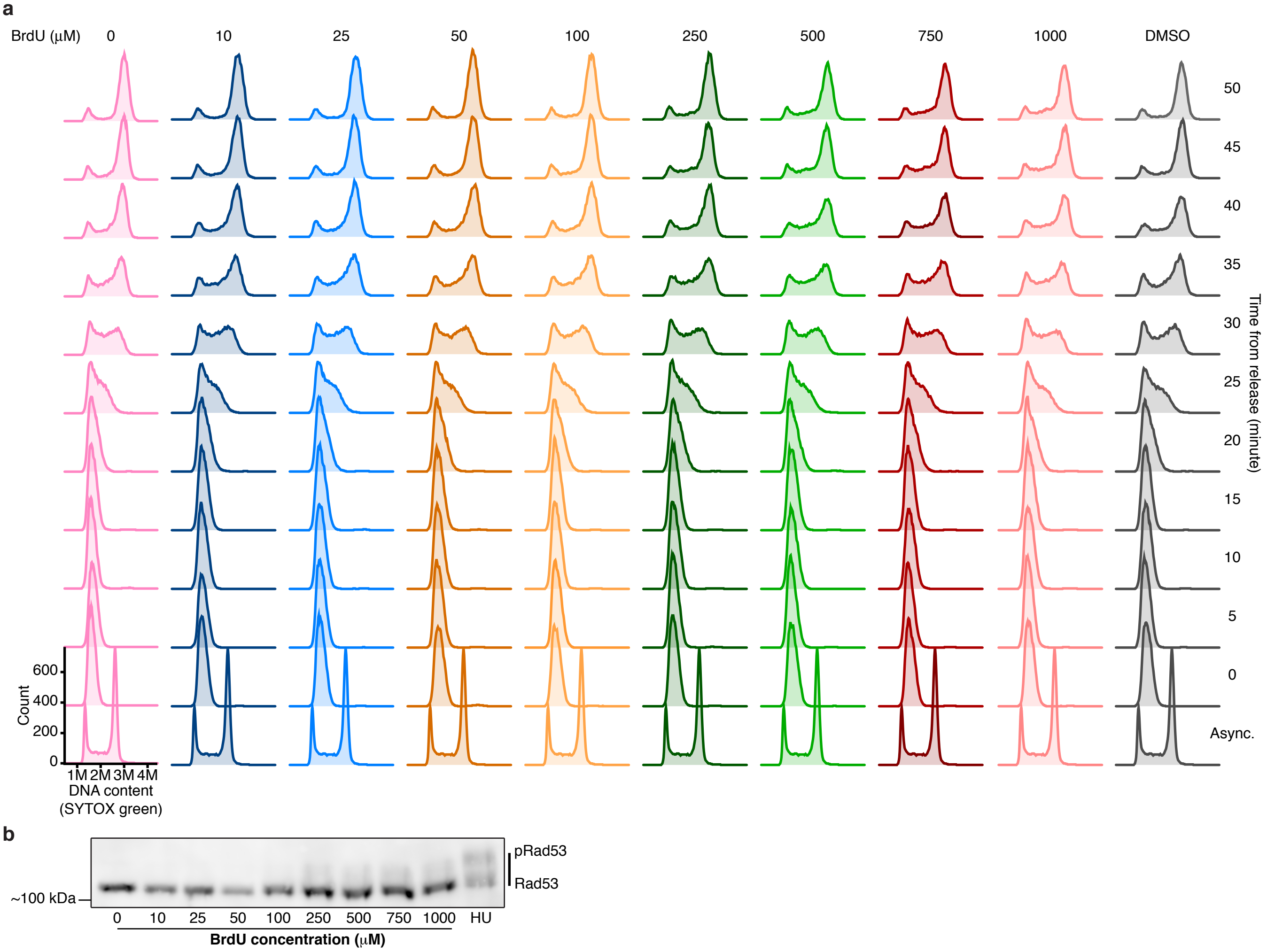

Supplementary Figure 5. Doses of BrdU up to 100 μM do not impact S phase progression nor activate Rad53 in BT1 cells. a, Representative FACS analysis of S phase progression of BT1 cells in the presence of increasing doses of BrdU. BT1 cells were arrested in G1 with α-factor, released synchronously into S phase and added with BrdU after 15 min. This experiment was performed twice independently with similar results. Async., asynchronous. **b**, Representative western blot analysis with anti-Rad53 antibodies of extracts from exponentially growing BT1 cells treated with increasing doses of BrdU for 30 min. Retarded bands correspond to phosphorylated (activated) Rad53, which exhibit a slower mobility than unphosphorylated Rad53. Cells treated with 200 mM hydroxyurea (HU) were used as a positive control for Rad53 phosphorylation. This experiment was performed twice independently with similar results. Uncropped blot is available in Source Data.

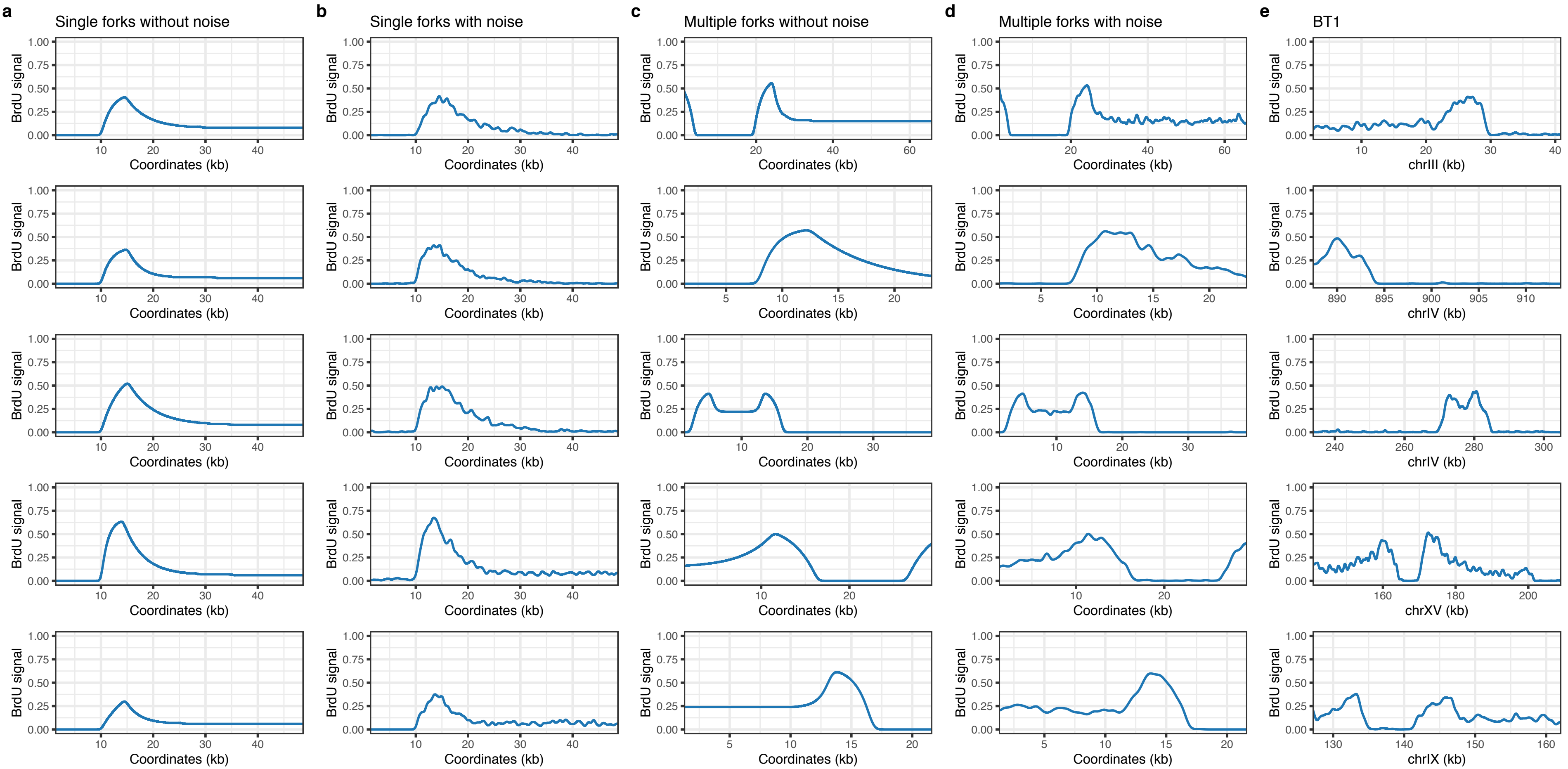

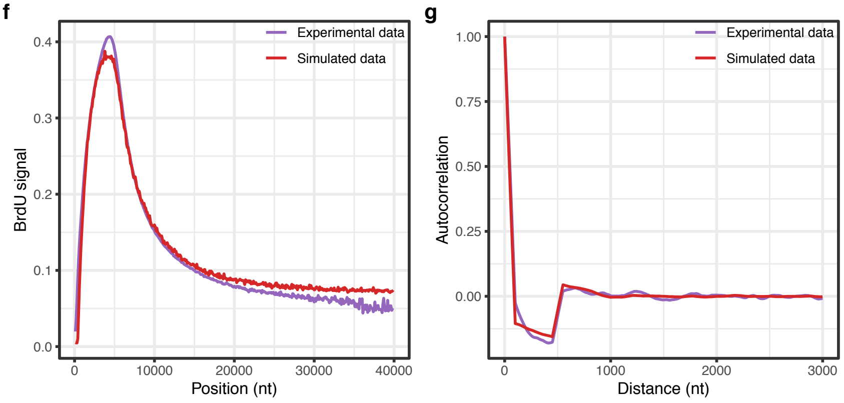

Supplementary Figure 6. Comparison of experimental and simulated reads. a-e, Examples of simulated reads containing a single fork (**a**, **b**) or one or multiple forks (**c**, **d**) without (**a**, **c**) or with (**b**, **d**) noise. BrdU content profiles of experimental nanopore reads from pulse-labelled BT1 cells (smoothed data after Megalodon processing) are shown in (**e**) for comparison. Visual inspection shows that forks from authentic and simulated reads with noise are indistinguishable. Simulated and experimental reads shown here were randomly selected. **f**, Mean BrdU traces of oriented replication signals from experimental (purple curve) and simulated data (red curve). **g**, Autocorrelation of the mean BrdU signal of experimental and simulated forks. Autocorrelation allows the determination of the scale at which the noise at one position of the signal is correlated to the noise at a given distance. Autocorrelation of the mean BrdU signal dropped to 0 very fast (*i.e.*, within ≈ 100 bp) for both the experimental and simulated data (purple and red curve, respectively), indicating that the noise added to the simulated forks resembles the experimental one. See Methods for details.

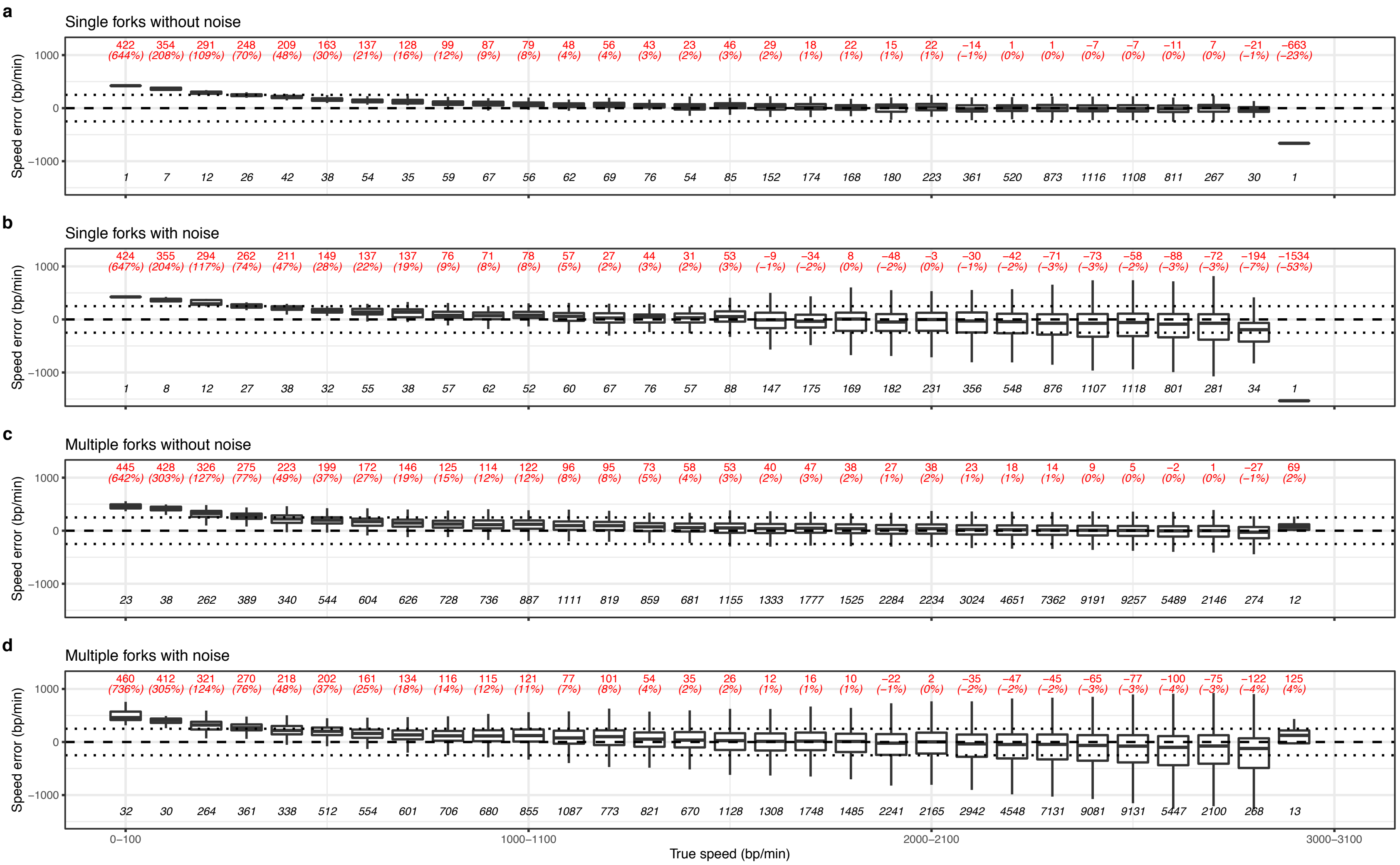

Supplementary Figure 7. Distribution of measurement errors made by NFS according to fork speed. a-d, Fork velocities were determined by NFS on 10,000 simulated reads containing a single fork of known speed without (**a**) or with (**b**) noise and on 100,000 simulated reads harbouring one or multiple forks without (**c**) or with (**d**) noise, and speed errors (*i.e.*, the difference between NFS measurement and the actual speed of a given fork) were grouped according to fork speed sorted into 100 bp/min categories (0-100, 100-200, etc) and represented as boxplots. Thick horizontal black line, median speed error, value indicated in red on top, with the median relative speed error indicated below; box lower and upper hinges, 1st and 3rd quartiles, respectively; upper and lower whiskers, largest and smallest value no further than 1.5x interquartile range from the hinge, respectively; the number of values is indicated below each box. Horizontal dotted lines at speed errors of -250, 0 and 250 bp/min are here to guide the eyes. Forks were simulated on the basis of our estimate of the true fork speed distribution in yeast (see Methods).

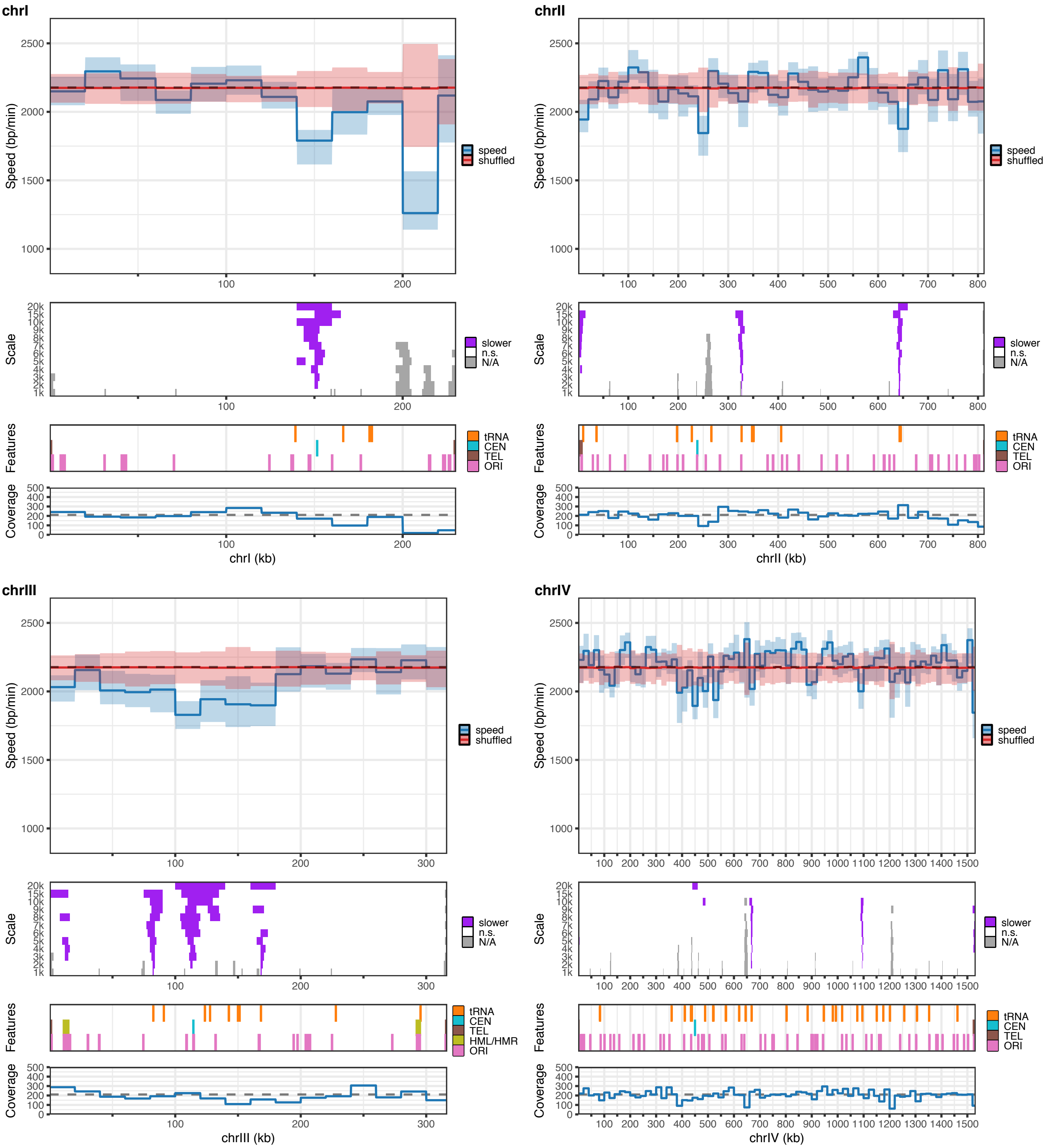

Supplementary Figure 8. Replication fork progression map of yeast chromosomes I to X, XV and XVI. See Fig. 5 caption for details.

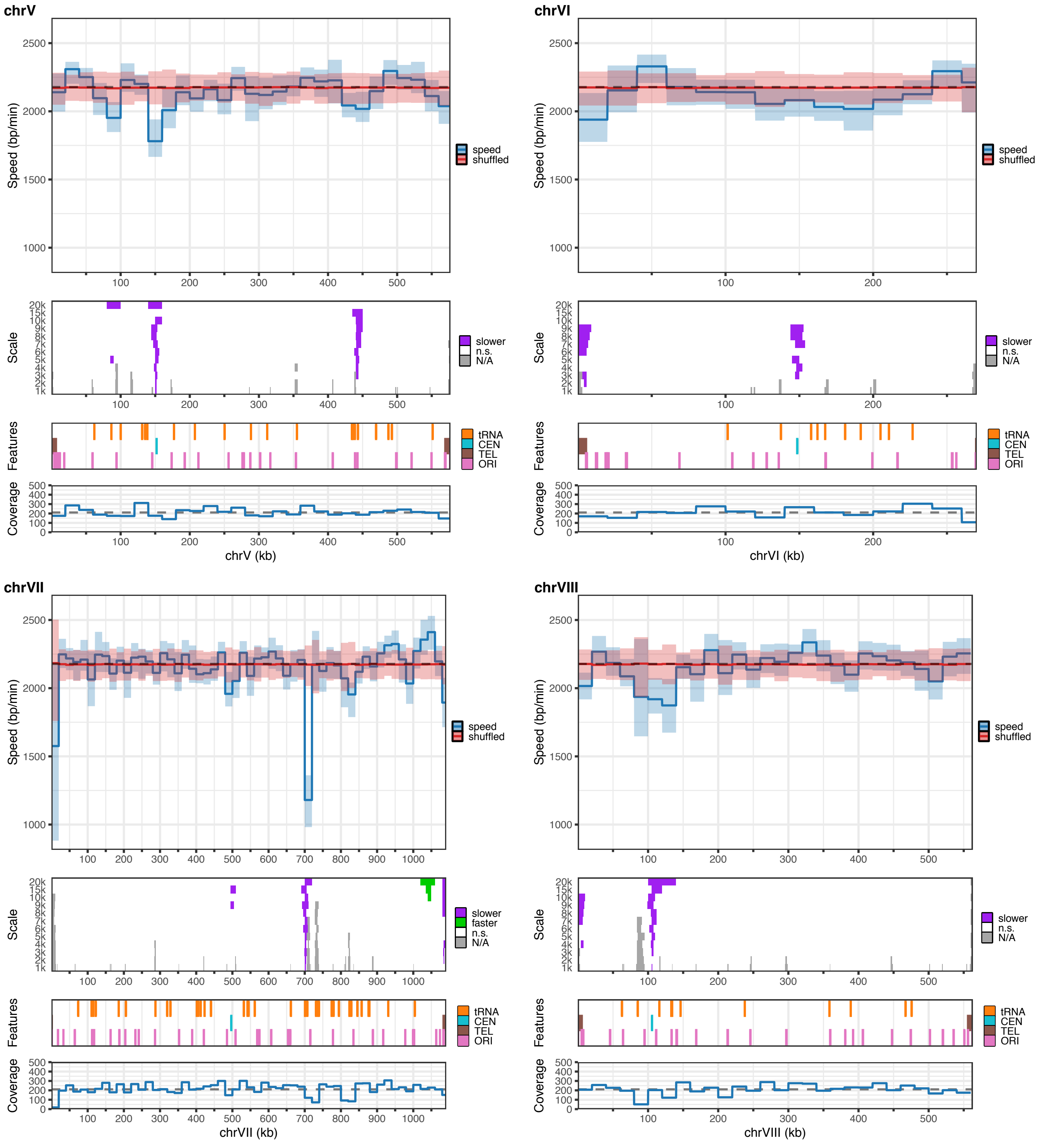

Supplementary Figure 8, continued.

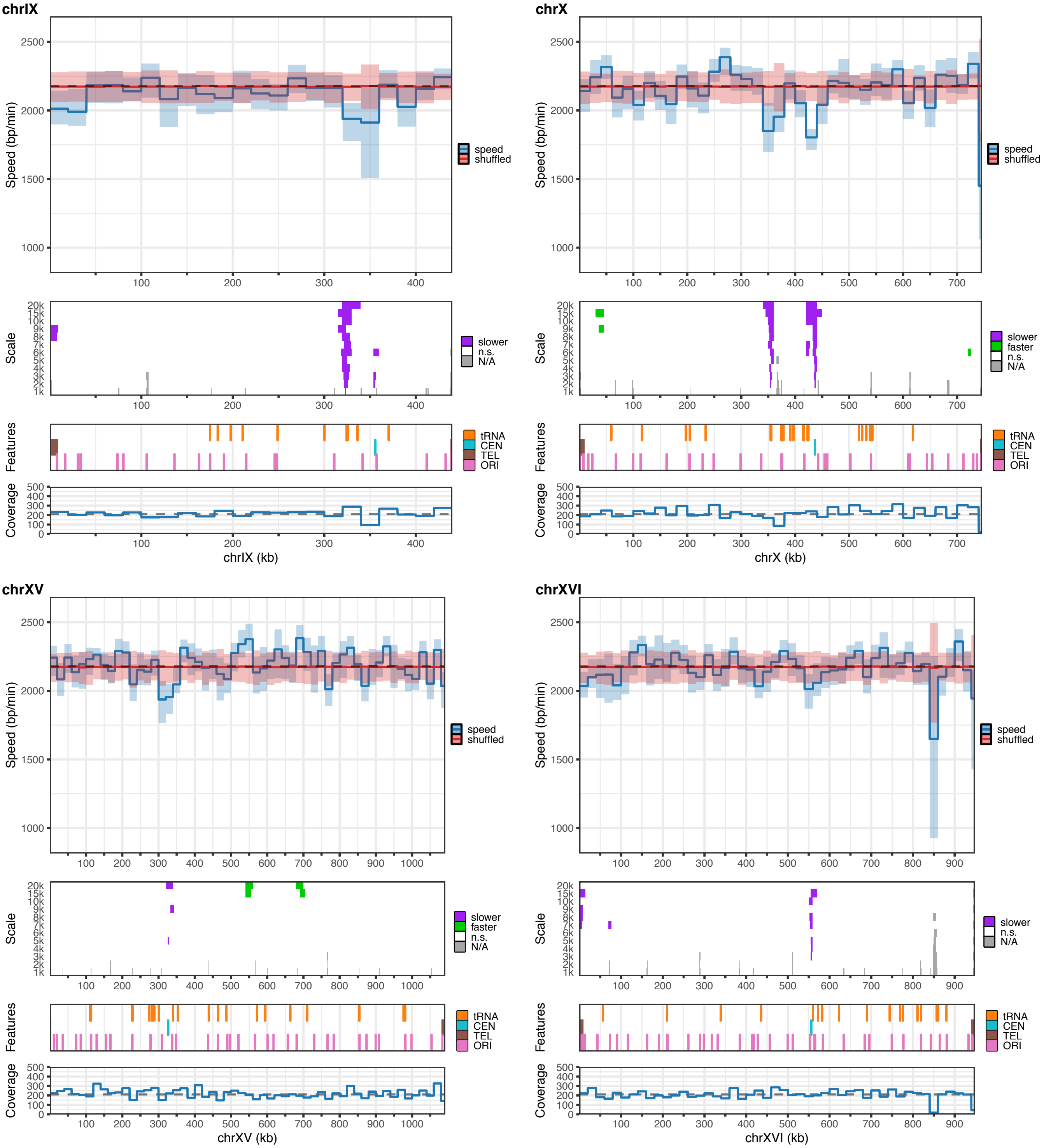

Supplementary Figure 8, continued.

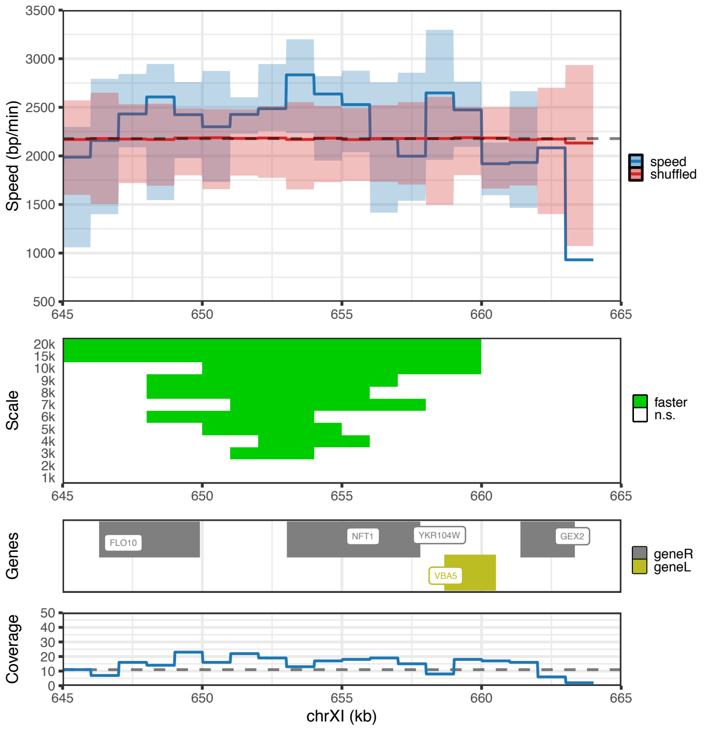

Supplementary Figure 9. Replication fork progression map of a portion of chromosome XI. Shown is the region of chromosome XI where forks seemingly travel faster than in the rest of the genome. Panels from top to bottom: 1) median of experimental fork speeds (blue line) with 98% confidence interval of the median (light blue) and median of reshuffled speeds (red line) with 98% confidence interval of the median (light red) computed in 1 kb windows (dotted line, median fork speed in the whole genome); 2) results of Mann-Whitney-Wilcoxon tests with Holm correction (one-sided) performed along the chromosome to compare the speed distribution in a given window of a given width (1, 2, 3, 4, 5, 6, 7, 8, 9, 10, 15 and 20 kb) to the speed distribution on the whole genome (green, regions of higher fork speed; white, n.s., not significant; statistical significance was set to $p < 0.01$); 3) position of genes (geneR and geneL, rightward and leftward direction of transcription, respectively); 4) coverage of individual replication fork velocities (dotted line, median coverage of the genome). No tRNA nor replication origin (from[19]) are present in this region. Please note that it was not possible to compute a finite confidence interval for the median of the experimental fork speeds for windows with a coverage ≤ 5.

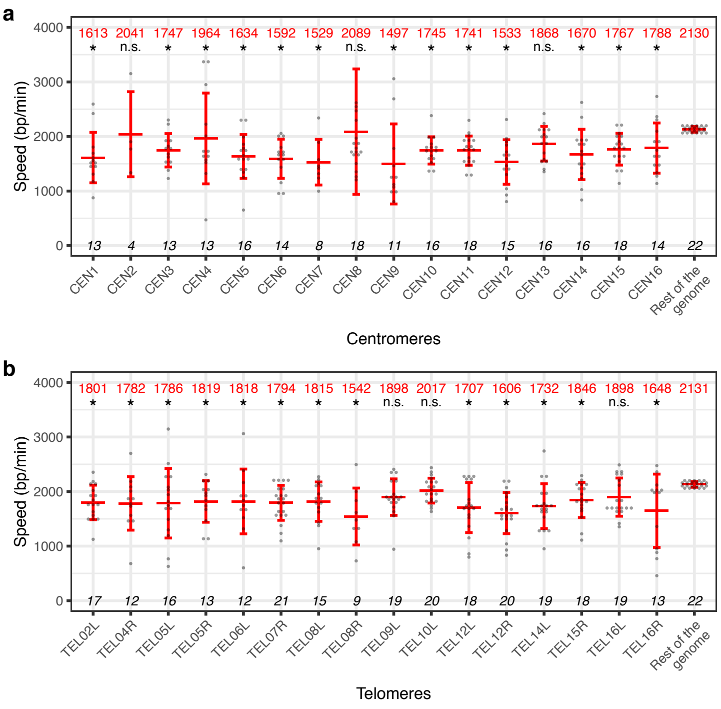

Supplementary Figure 10. Targeted analysis of replication fork progression at individual centromeres and telomeres. a, **b**, Replication fork speed at individual centromeres (**a**) or telomeres (**b**). See Fig. 6 caption for details. The number of samples varies because no overlapping fork was detected at certain centromeres or telomeres in some samples; please note that (i) telomeres with no overlapping fork in any of the samples are not represented, (ii) «telomeres» correspond to subtelomeric sequences and do not comprise the terminal stretch of telomeric repeats and (iii) one sample, with a value of 6,384 bp/min, is out of bounds for CEN8. Two-sided contrast comparisons between a given centromere (**a**) or telomere (**b**) and the rest of the genome are indicated by a star ($p \leq 0.05$) or n.s. (not significant). Statistical analyses are detailed in Supplementary Table 2. CEN#, centromere of the indicated chromosome; TEL#L/R, left (L) or right (R) telomere of the indicated chromosome.

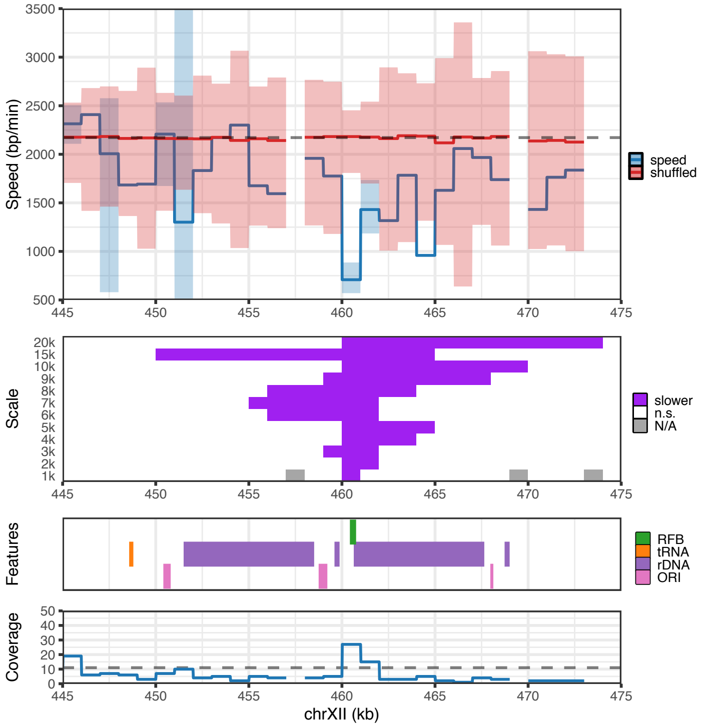

Supplementary Figure 11. Replication fork progression map of a portion of chromosome XII containing the rDNA locus. Panels from top to bottom: 1) median of experimental fork speeds (blue line) with 98% confidence interval of the median (light blue) and median of reshuffled speeds (red line) with 98% confidence interval of the median (light red) computed in 1 kb windows (dotted line, median fork speed in the whole genome); 2) results of Mann-Whitney-Wilcoxon tests with Holm correction (one-sided) performed along the chromosome to compare the speed distribution in a given window of a given width (1, 2, 3, 4, 5, 6, 7, 8, 9, 10, 15 and 20 kb) to the speed distribution on the whole genome (purple, regions of lower fork speed; white, n.s., not significant; statistical significance was set to $p < 0.01$; grey, N/A, not applicable, regions with no fork); 3) position of selected genomic features (large and short horizontal light purple bars, 35S and 5S ribosomal genes, respectively; please note that the rDNA locus is composed of two rDNA units in the sacCer3 version of the yeast genome used in this study; RFB, replication fork barrier; ORI, known *S. cerevisiae* replication origins from[21]); 4) coverage of individual replication fork velocities (dotted line, median coverage of the genome). Please note that it was not possible to compute a finite confidence interval for the median of the experimental fork speeds for windows with a coverage ≤ 5.

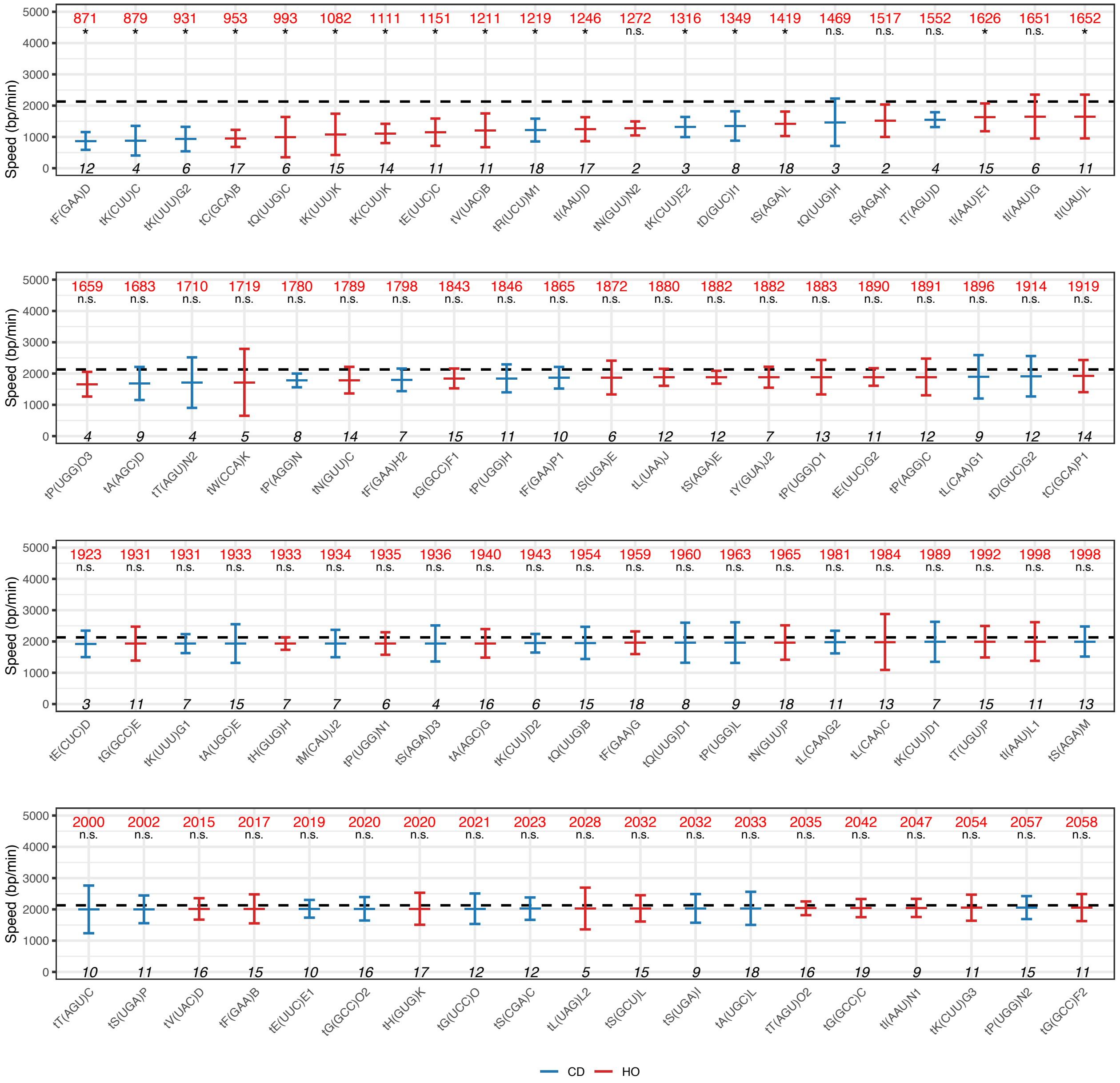

Supplementary Figure 12. Targeted analysis of replication fork progression at individual tRNA genes. The speed of forks overlapping individual tRNA genes was computed. The name of each tRNA gene is displayed below each plot; the predominant direction of replication relative to transcription (CD, co-directional, in blue; HO, head-on, in red; see Methods) is specified for every tRNA gene. tRNA genes are ordered according to increasing average fork speed. Centre line, average value of the mean speeds of forks overlapping the indicated tRNA gene in each sample, indicated in red on top (please note that individual data points are not shown for clarity); whiskers, standard deviation; bottom, number of samples (this number varies as no overlapping fork was detected at certain tRNA genes in some samples; please note that tRNA genes with no overlapping fork in any of the samples as well as tRNA genes with overlapping forks detected in only one sample, hence for which no contrast test could be performed, are not represented); dotted line at 2,130 bp/min, average fork speed of the 22 "Rest of the genome" samples. Two-sided contrast comparisons between a given tRNA gene and the rest of the genome are indicated by a star ($p \leq 0.05$) or n.s. (not significant). Statistical analyses are detailed in Supplementary Table 2.

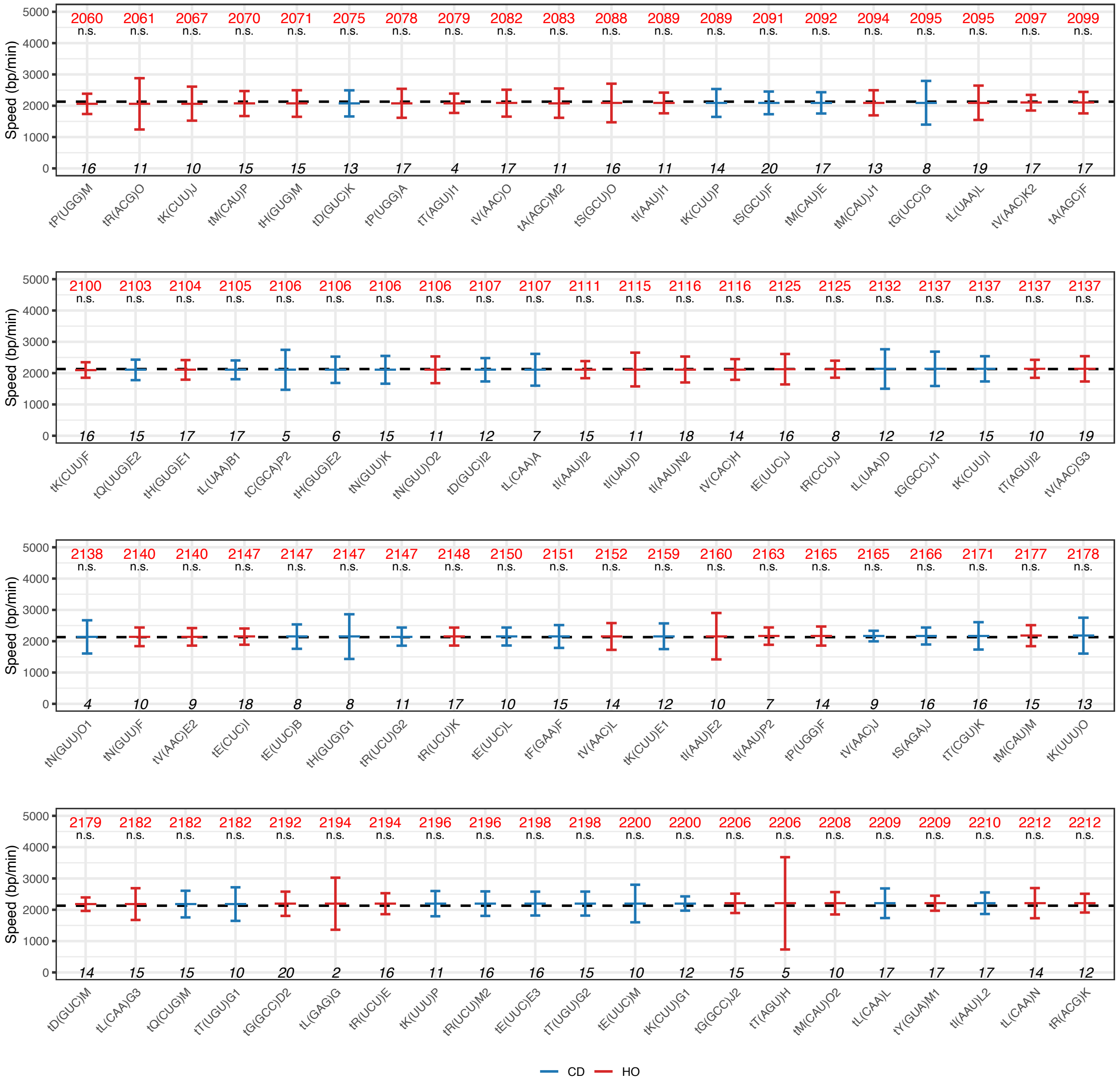

Supplementary Figure 12, continued.

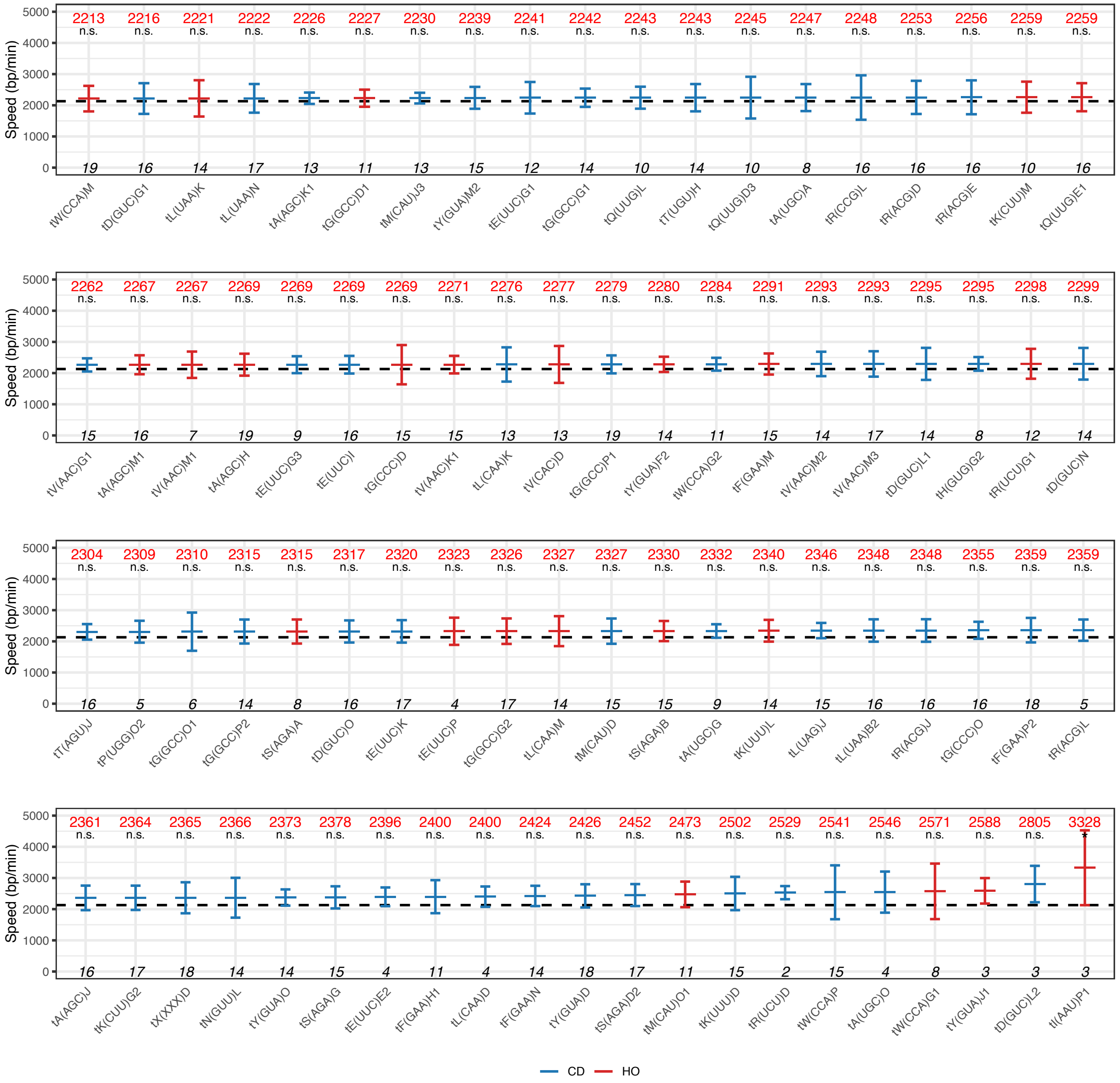

Supplementary Figure 12, continued.

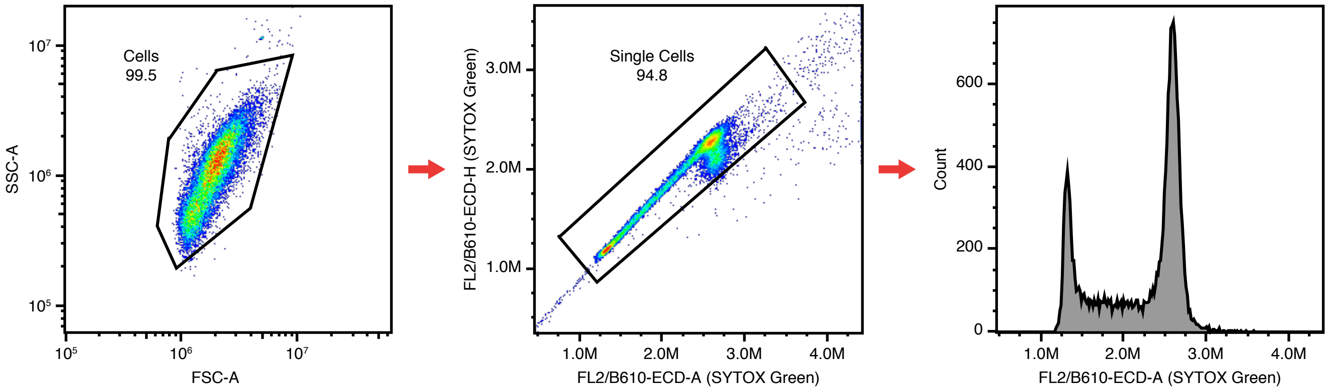

Supplementary Figure 13. Gating strategy for flow cytometry analysis. Cells are fixed in ethanol and DNA is counterstained with SYTOX Green prior to flow cytometry analysis. Cells are initially gated using the FSC-Area versus SSC-Area plot to remove debris (left panel), then interrogated by the ratios of area (FL2/B610-ECD-A) to height (FL2/B610-ECD-H) of the SYTOX Green signal to gate out cell doublets (middle panel). A histogram of the FL2/B610-ECD-Area (right panel) shows DNA content after gating.

Supplementary Table 1. Strains and plasmids used in this study. CO, codon-optimized for expression in yeast.

Yeast strain	Genotype	Reference
W303 (*URA3 bar1Δ*)	*MATa trp1-1 leu2-3,112 his3-11,15 bar1::LEU2*	This study
BT1	*MATa trp1-1 leu2-3,112 his3-11,15 bar1::LEU2 ura3-1::URA3-GPD-hsvTK$_{CO}$-ADH-hENT1$_{CO}$(5x)*	This study
BT2	*MATa ade2-1 trp1-1 leu2-3,112 his3-11,15 can1-100 bar1::LEU2 ura3-1::URA3-GPD-hsvTK(7x) aur1::AUR1C-ADH-hENT1$_{CO}$(3x)*	This study
BT3	*MATa trp1-1 leu2-3,112 his3-11,15 bar1::LEU2 ura3-1::URA3-GPD-hsvTK-ADH-hENT1(5x)*	This study
BT4	BT1 *rtt109::KANMX*	This study
BT5	BT1 *sml1::KANMX*	This study
BT6	BT1 *csm3::KANMX*	This study
BT7	BT1 *tof1::KANMX*	This study
BT8	BT1 *mrc1::KANMX*	This study
MCM869	*MATa ade2-1 trp1-1 leu2-3,112 his3-11,15 can1-100 bar1::LEU2 ura3-1::URA3-GPD-hsvTK(7x) aur1::AUR1C-ADH-hENT1$_{CO}$(3x) cdc21::KANMX*	Ma et al, 2012
**Plasmid**	**Description**	**Reference**
p306-BrdU-Inc	Integrating plasmid for incorporation of exogenous thymidine or thymidine analogs	Viggiani et al, 2006
pBL-hsvTK$_{CO}$-hENT1$_{CO}$	Integrating plasmid for incorporation of exogenous thymidine or thymidine analogs (transgenes codon-optimized for expression in yeast)	This study

Supplementary Table 2. Statistical analyses. "other", rest of the genome; n, sample size for each class tested; df, degree of freedom; nr, not relevant; adjusted p values from the same panel, according to Benjamini & Hochberg.

Figure	Comparison	Alternative hypothesis	Sample size	Test	p-value	Adjusted p-value
4A	0 and 1	effect difference	n = 22, n = 2 | df = 29	Contrast test in linear model	1e-4	1e-4
	0 and 2.5	effect difference	n = 22, n = 2 | df = 29	Contrast test in linear model	2e-15	2e-15
	0 and 5	effect difference	n = 22, n = 2 | df = 29	Contrast test in linear model	4e-18	6e-18
	0 and 10	effect difference	n = 22, n = 2 | df = 29	Contrast test in linear model	8e-21	1e-20
	0 and 25	effect difference	n = 22, n = 2 | df = 29	Contrast test in linear model	1e-26	3e-26
	0 and 50	effect difference	n = 22, n = 2 | df = 29	Contrast test in linear model	1e-28	5e-28
	0 and 100	effect difference	n = 22, n = 2 | df = 29	Contrast test in linear model	4e-31	3e-30
4B	BT1 and csm3	effect difference	n = 22, n = 2 | df = 26	Contrast test in linear model	1e-32	2e-32
	BT1 and mrc1	effect difference	n = 22, n = 2 | df = 26	Contrast test in linear model	4e-42	2e-41
	BT1 and rtt109	effect difference	n = 22, n = 2 | df = 26	Contrast test in linear model	4e-24	5e-24
	BT1 and sml1	effect difference	n = 22, n = 2 | df = 26	Contrast test in linear model	8e-21	8e-21
	BT1 and tof1	effect difference	n = 22, n = 2 | df = 26	Contrast test in linear model	2e-33	4e-33
6A	other and centromere	effect difference	n = 22, n = 22 | df = 81	Contrast test in linear model	1e-4	3e-4
	other and rDNA	effect difference	n = 22, n = 19 | df = 81	Contrast test in linear model	1e-11	5e-11
	other and telomere	effect difference	n = 22, n = 22 | df = 81	Contrast test in linear model	8e-4	0.001
	other and tRNA	effect difference	n = 22, n = 22 | df = 81	Contrast test in linear model	0.77	0.77
6B	CD and HO	effect difference	n = 22, n = 22 | df = 21	Contrast test in linear model	3e-8	*nr*
6C	Leading and lagging	effect difference	n = 22, n = 22 | df = 21	Contrast test in linear model	0.04	*nr*
S10A	other and CEN1	effect difference	n = 22, n = 13 | df = 207	Contrast test in linear model	6e-4	2e-3
	other and CEN2	effect difference	n = 22, n = 4 | df = 207	Contrast test in linear model	0.41	0.41
	other and CEN3	effect difference	n = 22, n = 13 | df = 207	Contrast test in linear model	0.02	0.03
	other and CEN4	effect difference	n = 22, n = 13 | df = 207	Contrast test in linear model	0.03	0.04
	other and CEN5	effect difference	n = 22, n = 16 | df = 207	Contrast test in linear model	5e-4	0.002
	other and CEN6	effect difference	n = 22, n = 14 | df = 207	Contrast test in linear model	9e-4	0.002
	other and CEN7	effect difference	n = 22, n = 8 | df = 207	Contrast test in linear model	0.005	0.01
	other and CEN8	effect difference	n = 22, n = 18 | df = 207	Contrast test in linear model	0.29	0.31
	other and CEN9	effect difference	n = 22, n = 11 | df = 207	Contrast test in linear model	2e-5	4e-4
	other and CEN10	effect difference	n = 22, n = 16 | df = 207	Contrast test in linear model	0.03	0.04
	other and CEN11	effect difference	n = 22, n = 18 | df = 207	Contrast test in linear model	0.01	0.02
	other and CEN12	effect difference	n = 22, n = 15 | df = 207	Contrast test in linear model	6e-5	5e-4
	other and CEN13	effect difference	n = 22, n = 16 | df = 207	Contrast test in linear model	0.09	0.10
	other and CEN14	effect difference	n = 22, n = 16 | df = 207	Contrast test in linear model	5e-4	2e-3
	other and CEN15	effect difference	n = 22, n = 18 | df = 207	Contrast test in linear model	0.02	0.03
	other and CEN16	effect difference	n = 22, n = 14 | df = 207	Contrast test in linear model	0.02	0.03
S10B	other and TEL02L	effect difference	n = 22, n = 17 | df = 245	Contrast test in linear model	0.03	0.04
	other and TEL04R	effect difference	n = 22, n = 12 | df = 245	Contrast test in linear model	0.008	0.02
	other and TEL05L	effect difference	n = 22, n = 16 | df = 245	Contrast test in linear model	0.006	0.02
	other and TEL05R	effect difference	n = 22, n = 13 | df = 245	Contrast test in linear model	0.03	0.04
	other and TEL06L	effect difference	n = 22, n = 12 | df = 245	Contrast test in linear model	0.02	0.03
	other and TEL07R	effect difference	n = 22, n = 21 | df = 245	Contrast test in linear model	0.01	0.03
	other and TEL08L	effect difference	n = 22, n = 15 | df = 245	Contrast test in linear model	0.02	0.03
	other and TEL08R	effect difference	n = 22, n = 9 | df = 245	Contrast test in linear model	2e-4	0.001
	other and TEL09L	effect difference	n = 22, n = 19 | df = 245	Contrast test in linear model	0.10	0.11
	other and TEL10L	effect difference	n = 22, n = 20 | df = 245	Contrast test in linear model	0.43	0.43
	other and TEL12L	effect difference	n = 22, n = 18 | df = 245	Contrast test in linear model	0.001	0.005
	other and TEL12R	effect difference	n = 22, n = 20 | df = 245	Contrast test in linear model	9e-5	8e-4
	other and TEL14L	effect difference	n = 22, n = 19 | df = 245	Contrast test in linear model	0.004	0.01
	other and TEL15R	effect difference	n = 22, n = 18 | df = 245	Contrast test in linear model	0.03	0.04
	other and TEL16L	effect difference	n = 22, n = 19 | df = 245	Contrast test in linear model	0.10	0.11
	other and TEL16R	effect difference	n = 22, n = 13 | df = 245	Contrast test in linear model	9e-5	8e-4

Figure	Comparison	Alternative hypothesis	Sample size	Test	p-value	Adjusted p-value
	other and tA(AGC)D	effect difference	n = 22, n = 9 | df = 2728	Contrast test in linear model	0.01	0.13
	other and tA(AGC)F	effect difference	n = 22, n = 17 | df = 2728	Contrast test in linear model	0.82	0.97
	other and tA(AGC)G	effect difference	n = 22, n = 16 | df = 2728	Contrast test in linear model	0.19	0.68
	other and tA(AGC)H	effect difference	n = 22, n = 19 | df = 2728	Contrast test in linear model	0.30	0.78
	other and tA(AGC)J	effect difference	n = 22, n = 16 | df = 2728	Contrast test in linear model	0.11	0.55
	other and tA(AGC)K1	effect difference	n = 22, n = 13 | df = 2728	Contrast test in linear model	0.53	0.86
	other and tA(AGC)M1	effect difference	n = 22, n = 16 | df = 2728	Contrast test in linear model	0.35	0.78
	other and tA(AGC)M2	effect difference	n = 22, n = 11 | df = 2728	Contrast test in linear model	0.81	0.97
	other and tA(UGC)A	effect difference	n = 22, n = 8 | df = 2728	Contrast test in linear model	0.55	0.87
	other and tA(UGC)E	effect difference	n = 22, n = 15 | df = 2728	Contrast test in linear model	0.18	0.65
	other and tA(UGC)G	effect difference	n = 22, n = 9 | df = 2728	Contrast test in linear model	0.23	0.72
	other and tA(UGC)L	effect difference	n = 22, n = 18 | df = 2728	Contrast test in linear model	0.50	0.84
	other and tA(UGC)O	effect difference	n = 22, n = 4 | df = 2728	Contrast test in linear model	0.08	0.50
	other and tC(GCA)B	effect difference	n = 22, n = 17 | df = 2728	Contrast test in linear model	2e-16	5e-14
	other and tC(GCA)P1	effect difference	n = 22, n = 14 | df = 2728	Contrast test in linear model	0.16	0.64
	other and tC(GCA)P2	effect difference	n = 22, n = 5 | df = 2728	Contrast test in linear model	0.95	0.97
	other and tD(GUC)G1	effect difference	n = 22, n = 16 | df = 2728	Contrast test in linear model	0.55	0.88
	other and tD(GUC)G2	effect difference	n = 22, n = 12 | df = 2728	Contrast test in linear model	0.17	0.65
	other and tD(GUC)I1	effect difference	n = 22, n = 8 | df = 2728	Contrast test in linear model	2e-5	4e-4
	other and tD(GUC)I2	effect difference	n = 22, n = 12 | df = 2728	Contrast test in linear model	0.88	0.97
	other and tD(GUC)K	effect difference	n = 22, n = 13 | df = 2728	Contrast test in linear model	0.73	0.95
	other and tD(GUC)L1	effect difference	n = 22, n = 14 | df = 2728	Contrast test in linear model	0.27	0.76
	other and tD(GUC)L2	effect difference	n = 22, n = 3 | df = 2728	Contrast test in linear model	0.01	0.14
	other and tD(GUC)M	effect difference	n = 22, n = 14 | df = 2728	Contrast test in linear model	0.77	0.97
	other and tD(GUC)N	effect difference	n = 22, n = 14 | df = 2728	Contrast test in linear model	0.25	0.73
	other and tD(GUC)O	effect difference	n = 22, n = 16 | df = 2728	Contrast test in linear model	0.20	0.68
	other and tE(CUC)D	effect difference	n = 22, n = 3 | df = 2728	Contrast test in linear model	0.44	0.82
	other and tE(CUC)I	effect difference	n = 22, n = 18 | df = 2728	Contrast test in linear model	0.90	0.97
	other and tE(UUC)B	effect difference	n = 22, n = 8 | df = 2728	Contrast test in linear model	0.97	0.98
	other and tE(UUC)C	effect difference	n = 22, n = 11 | df = 2728	Contrast test in linear model	2e-9	6e-8
	other and tE(UUC)E1	effect difference	n = 22, n = 10 | df = 2728	Contrast test in linear model	0.53	0.86
	other and tE(UUC)E2	effect difference	n = 22, n = 4 | df = 2728	Contrast test in linear model	0.26	0.74
	other and tE(UUC)E3	effect difference	n = 22, n = 16 | df = 2728	Contrast test in linear model	0.63	0.92
	other and tE(UUC)G1	effect difference	n = 22, n = 12 | df = 2728	Contrast test in linear model	0.49	0.84
	other and tE(UUC)G2	effect difference	n = 22, n = 11 | df = 2728	Contrast test in linear model	0.14	0.59
	other and tE(UUC)G3	effect difference	n = 22, n = 9 | df = 2728	Contrast test in linear model	0.42	0.82
	other and tE(UUC)I	effect difference	n = 22, n = 16 | df = 2728	Contrast test in linear model	0.33	0.78
	other and tE(UUC)J	effect difference	n = 22, n = 16 | df = 2728	Contrast test in linear model	0.97	0.98
S12	other and tE(UUC)K	effect difference	n = 22, n = 17 | df = 2728	Contrast test in linear model	0.18	0.65
	other and tE(UUC)L	effect difference	n = 22, n = 10 | df = 2728	Contrast test in linear model	0.90	0.97
	other and tE(UUC)M	effect difference	n = 22, n = 10 | df = 2728	Contrast test in linear model	0.69	0.94
	other and tE(UUC)P	effect difference	n = 22, n = 4 | df = 2728	Contrast test in linear model	0.41	0.82
	other and tF(GAA)B	effect difference	n = 22, n = 15 | df = 2728	Contrast test in linear model	0.44	0.82
	other and tF(GAA)D	effect difference	n = 22, n = 12 | df = 2728	Contrast test in linear model	2e-15	3e-13
	other and tF(GAA)F	effect difference	n = 22, n = 15 | df = 2728	Contrast test in linear model	0.90	0.97
	other and tF(GAA)G	effect difference	n = 22, n = 18 | df = 2728	Contrast test in linear model	0.23	0.72
	other and tF(GAA)H1	effect difference	n = 22, n = 11 | df = 2728	Contrast test in linear model	0.09	0.53
	other and tF(GAA)H2	effect difference	n = 22, n = 7 | df = 2728	Contrast test in linear model	0.09	0.53
	other and tF(GAA)M	effect difference	n = 22, n = 15 | df = 2728	Contrast test in linear model	0.28	0.78
	other and tF(GAA)N	effect difference	n = 22, n = 14 | df = 2728	Contrast test in linear model	0.05	0.39
	other and tF(GAA)P1	effect difference	n = 22, n = 10 | df = 2728	Contrast test in linear model	0.13	0.57
	other and tF(GAA)P2	effect difference	n = 22, n = 18 | df = 2728	Contrast test in linear model	0.10	0.54
	other and tG(CCC)D	effect difference	n = 22, n = 15 | df = 2728	Contrast test in linear model	0.36	0.78
	other and tG(CCC)O	effect difference	n = 22, n = 16 | df = 2728	Contrast test in linear model	0.12	0.57
	other and tG(GCC)C	effect difference	n = 22, n = 19 | df = 2728	Contrast test in linear model	0.51	0.86
	other and tG(GCC)D1	effect difference	n = 22, n = 11 | df = 2728	Contrast test in linear model	0.57	0.89
	other and tG(GCC)D2	effect difference	n = 22, n = 20 | df = 2728	Contrast test in linear model	0.65	0.92
	other and tG(GCC)E	effect difference	n = 22, n = 11 | df = 2728	Contrast test in linear model	0.22	0.72
	other and tG(GCC)F1	effect difference	n = 22, n = 15 | df = 2728	Contrast test in linear model	0.05	0.39
	other and tG(GCC)F2	effect difference	n = 22, n = 11 | df = 2728	Contrast test in linear model	0.66	0.93
	other and tG(GCC)G1	effect difference	n = 22, n = 14 | df = 2728	Contrast test in linear model	0.46	0.83
	other and tG(GCC)G2	effect difference	n = 22, n = 17 | df = 2728	Contrast test in linear model	0.16	0.64
	other and tG(GCC)J1	effect difference	n = 22, n = 12 | df = 2728	Contrast test in linear model	0.94	0.97
	other and tG(GCC)J2	effect difference	n = 22, n = 15 | df = 2728	Contrast test in linear model	0.61	0.91
	other and tG(GCC)O1	effect difference	n = 22, n = 6 | df = 2728	Contrast test in linear model	0.37	0.79
	other and tG(GCC)O2	effect difference	n = 22, n = 16 | df = 2728	Contrast test in linear model	0.46	0.83
	other and tG(GCC)P1	effect difference	n = 22, n = 19 | df = 2728	Contrast test in linear model	0.28	0.78
	other and tG(GCC)P2	effect difference	n = 22, n = 14 | df = 2728	Contrast test in linear model	0.21	0.71
	other and tG(UCC)G	effect difference	n = 22, n = 8 | df = 2728	Contrast test in linear model	0.86	0.97
	other and tG(UCC)O	effect difference	n = 22, n = 12 | df = 2728	Contrast test in linear model	0.47	0.84
	other and tH(GUG)E1	effect difference	n = 22, n = 17 | df = 2728	Contrast test in linear model	0.85	0.97
	other and tH(GUG)E2	effect difference	n = 22, n = 6 | df = 2728	Contrast test in linear model	0.94	0.97
	other and tH(GUG)G1	effect difference	n = 22, n = 8 | df = 2728	Contrast test in linear model	0.92	0.97
	other and tH(GUG)G2	effect difference	n = 22, n = 8 | df = 2728	Contrast test in linear model	0.33	0.78
	other and tH(GUG)H	effect difference	n = 22, n = 7 | df = 2728	Contrast test in linear model	0.30	0.78
	other and tH(GUG)K	effect difference	n = 22, n = 17 | df = 2728	Contrast test in linear model	0.44	0.82
	other and tH(GUG)M	effect difference	n = 22, n = 15 | df = 2728	Contrast test in linear model	0.71	0.95

Figure	Comparison	Alternative hypothesis	Sample size	Test	p-value	Adjusted p-value
	other and tI(AAU)D	effect difference	n = 22, n = 17 | df = 2728	Contrast test in linear model	6e-10	2e-8
	other and tI(AAU)E1	effect difference	n = 22, n = 15 | df = 2728	Contrast test in linear model	6e-4	0.01
	other and tI(AAU)E2	effect difference	n = 22, n = 10 | df = 2728	Contrast test in linear model	0.84	0.97
	other and tI(AAU)G	effect difference	n = 22, n = 6 | df = 2728	Contrast test in linear model	0.02	0.16
	other and tI(AAU)I1	effect difference	n = 22, n = 11 | df = 2728	Contrast test in linear model	0.82	0.97
	other and tI(AAU)I2	effect difference	n = 22, n = 15 | df = 2728	Contrast test in linear model	0.89	0.97
	other and tI(AAU)L1	effect difference	n = 22, n = 11 | df = 2728	Contrast test in linear model	0.42	0.82
	other and tI(AAU)L2	effect difference	n = 22, n = 17 | df = 2728	Contrast test in linear model	0.58	0.89
	other and tI(AAU)N1	effect difference	n = 22, n = 9 | df = 2728	Contrast test in linear model	0.64	0.92
	other and tI(AAU)N2	effect difference	n = 22, n = 18 | df = 2728	Contrast test in linear model	0.94	0.97
	other and tI(AAU)P1	effect difference	n = 22, n = 3 | df = 2728	Contrast test in linear model	8e-6	2e-4
	other and tI(AAU)P2	effect difference	n = 22, n = 7 | df = 2728	Contrast test in linear model	0.80	0.97
	other and tI(UAU)D	effect difference	n = 22, n = 11 | df = 2728	Contrast test in linear model	0.93	0.97
	other and tI(UAU)L	effect difference	n = 22, n = 11 | df = 2728	Contrast test in linear model	0.003	0.047
	other and tK(CUU)C	effect difference	n = 22, n = 4 | df = 2728	Contrast test in linear model	1e-7	3e-6
	other and tK(CUU)D1	effect difference	n = 22, n = 7 | df = 2728	Contrast test in linear model	0.49	0.84
	other and tK(CUU)D2	effect difference	n = 22, n = 6 | df = 2728	Contrast test in linear model	0.36	0.78
	other and tK(CUU)E1	effect difference	n = 22, n = 12 | df = 2728	Contrast test in linear model	0.85	0.97
	other and tK(CUU)E2	effect difference	n = 22, n = 3 | df = 2728	Contrast test in linear model	0.003	0.047
	other and tK(CUU)F	effect difference	n = 22, n = 16 | df = 2728	Contrast test in linear model	0.84	0.97
	other and tK(CUU)G1	effect difference	n = 22, n = 12 | df = 2728	Contrast test in linear model	0.64	0.92
	other and tK(CUU)G2	effect difference	n = 22, n = 17 | df = 2728	Contrast test in linear model	0.10	0.53
	other and tK(CUU)G3	effect difference	n = 22, n = 11 | df = 2728	Contrast test in linear model	0.64	0.92
	other and tK(CUU)I	effect difference	n = 22, n = 15 | df = 2728	Contrast test in linear model	0.94	0.97
	other and tK(CUU)J	effect difference	n = 22, n = 10 | df = 2728	Contrast test in linear model	0.71	0.95
	other and tK(CUU)K	effect difference	n = 22, n = 14 | df = 2728	Contrast test in linear model	2e-11	9e-10
	other and tK(CUU)M	effect difference	n = 22, n = 10 | df = 2728	Contrast test in linear model	0.42	0.82
	other and tK(CUU)P	effect difference	n = 22, n = 14 | df = 2728	Contrast test in linear model	0.76	0.97
	other and tK(UUU)D	effect difference	n = 22, n = 15 | df = 2728	Contrast test in linear model	0.01	0.13
	other and tK(UUU)G1	effect difference	n = 22, n = 7 | df = 2728	Contrast test in linear model	0.30	0.78
	other and tK(UUU)G2	effect difference	n = 22, n = 6 | df = 2728	Contrast test in linear model	4e-9	1e-7
	other and tK(UUU)K	effect difference	n = 22, n = 15 | df = 2728	Contrast test in linear model	2e-12	1e-10
	other and tK(UUU)L	effect difference	n = 22, n = 14 | df = 2728	Contrast test in linear model	0.15	0.63
	other and tK(UUU)O	effect difference	n = 22, n = 13 | df = 2728	Contrast test in linear model	0.74	0.96
	other and tK(UUU)P	effect difference	n = 22, n = 11 | df = 2728	Contrast test in linear model	0.67	0.94
	other and tL(CAA)A	effect difference	n = 22, n = 7 | df = 2728	Contrast test in linear model	0.89	0.97
	other and tL(CAA)C	effect difference	n = 22, n = 13 | df = 2728	Contrast test in linear model	0.33	0.78
	other and tL(CAA)D	effect difference	n = 22, n = 4 | df = 2728	Contrast test in linear model	0.24	0.73
S12 (continued)	other and tL(CAA)G1	effect difference	n = 22, n = 9 | df = 2728	Contrast test in linear model	0.18	0.65
	other and tL(CAA)G2	effect difference	n = 22, n = 11 | df = 2728	Contrast test in linear model	0.36	0.78
	other and tL(CAA)G3	effect difference	n = 22, n = 15 | df = 2728	Contrast test in linear model	0.73	0.95
	other and tL(CAA)K	effect difference	n = 22, n = 13 | df = 2728	Contrast test in linear model	0.36	0.78
	other and tL(CAA)L	effect difference	n = 22, n = 17 | df = 2728	Contrast test in linear model	0.57	0.89
	other and tL(CAA)M	effect difference	n = 22, n = 14 | df = 2728	Contrast test in linear model	0.20	0.68
	other and tL(CAA)N	effect difference	n = 22, n = 14 | df = 2728	Contrast test in linear model	0.57	0.89
	other and tL(GAG)G	effect difference	n = 22, n = 2 | df = 2728	Contrast test in linear model	0.79	0.97
	other and tL(UAA)B1	effect difference	n = 22, n = 17 | df = 2728	Contrast test in linear model	0.87	0.97
	other and tL(UAA)B2	effect difference	n = 22, n = 16 | df = 2728	Contrast test in linear model	0.14	0.58
	other and tL(UAA)D	effect difference	n = 22, n = 12 | df = 2728	Contrast test in linear model	0.98	0.98
	other and tL(UAA)J	effect difference	n = 22, n = 12 | df = 2728	Contrast test in linear model	0.12	0.56
	other and tL(UAA)K	effect difference	n = 22, n = 14 | df = 2728	Contrast test in linear model	0.54	0.87
	other and tL(UAA)L	effect difference	n = 22, n = 19 | df = 2728	Contrast test in linear model	0.79	0.97
	other and tL(UAA)N	effect difference	n = 22, n = 17 | df = 2728	Contrast test in linear model	0.52	0.86
	other and tL(UAG)J	effect difference	n = 22, n = 15 | df = 2728	Contrast test in linear model	0.14	0.59
	other and tL(UAG)L2	effect difference	n = 22, n = 5 | df = 2728	Contrast test in linear model	0.68	0.94
	other and tM(CAU)D	effect difference	n = 22, n = 15 | df = 2728	Contrast test in linear model	0.17	0.65
	other and tM(CAU)E	effect difference	n = 22, n = 17 | df = 2728	Contrast test in linear model	0.81	0.97
	other and tM(CAU)J1	effect difference	n = 22, n = 13 | df = 2728	Contrast test in linear model	0.81	0.97
	other and tM(CAU)J2	effect difference	n = 22, n = 7 | df = 2728	Contrast test in linear model	0.31	0.78
	other and tM(CAU)J3	effect difference	n = 22, n = 13 | df = 2728	Contrast test in linear model	0.52	0.86
	other and tM(CAU)M	effect difference	n = 22, n = 15 | df = 2728	Contrast test in linear model	0.74	0.96
	other and tM(CAU)O1	effect difference	n = 22, n = 11 | df = 2728	Contrast test in linear model	0.03	0.29
	other and tM(CAU)O2	effect difference	n = 22, n = 10 | df = 2728	Contrast test in linear model	0.65	0.92
	other and tM(CAU)P	effect difference	n = 22, n = 15 | df = 2728	Contrast test in linear model	0.70	0.95
	other and tN(GUU)C	effect difference	n = 22, n = 14 | df = 2728	Contrast test in linear model	0.02	0.21
	other and tN(GUU)F	effect difference	n = 22, n = 10 | df = 2728	Contrast test in linear model	0.91	0.97
	other and tN(GUU)K	effect difference	n = 22, n = 15 | df = 2728	Contrast test in linear model	0.87	0.97
	other and tN(GUU)L	effect difference	n = 22, n = 14 | df = 2728	Contrast test in linear model	0.12	0.56
	other and tN(GUU)N2	effect difference	n = 22, n = 2 | df = 2728	Contrast test in linear model	0.01	0.13
	other and tN(GUU)O1	effect difference	n = 22, n = 4 | df = 2728	Contrast test in linear model	0.95	0.97
	other and tN(GUU)O2	effect difference	n = 22, n = 11 | df = 2728	Contrast test in linear model	0.87	0.97
	other and tN(GUU)P	effect difference	n = 22, n = 18 | df = 2728	Contrast test in linear model	0.24	0.73
	other and tP(AGG)C	effect difference	n = 22, n = 12 | df = 2728	Contrast test in linear model	0.13	0.58
	other and tP(AGG)N	effect difference	n = 22, n = 8 | df = 2728	Contrast test in linear model	0.05	0.39
	other and tP(UGG)A	effect difference	n = 22, n = 17 | df = 2728	Contrast test in linear model	0.72	0.95
	other and tP(UGG)F	effect difference	n = 22, n = 14 | df = 2728	Contrast test in linear model	0.82	0.97
	other and tP(UGG)H	effect difference	n = 22, n = 11 | df = 2728	Contrast test in linear model	0.09	0.53

Figure	Comparison	Alternative hypothesis	Sample size	Test	p-value	Adjusted p-value
	other and tP(UGG)L	effect difference	n = 22, n = 9 | df = 2728	Contrast test in linear model	0.35	0.78
	other and tP(UGG)M	effect difference	n = 22, n = 16 | df = 2728	Contrast test in linear model	0.62	0.92
	other and tP(UGG)N1	effect difference	n = 22, n = 6 | df = 2728	Contrast test in linear model	0.35	0.78
	other and tP(UGG)N2	effect difference	n = 22, n = 15 | df = 2728	Contrast test in linear model	0.61	0.91
	other and tP(UGG)O1	effect difference	n = 22, n = 13 | df = 2728	Contrast test in linear model	0.12	0.56
	other and tP(UGG)O2	effect difference	n = 22, n = 5 | df = 2728	Contrast test in linear model	0.39	0.81
	other and tP(UGG)O3	effect difference	n = 22, n = 4 | df = 2728	Contrast test in linear model	0.06	0.41
	other and tQ(CUG)M	effect difference	n = 22, n = 15 | df = 2728	Contrast test in linear model	0.70	0.95
	other and tQ(UUG)B	effect difference	n = 22, n = 15 | df = 2728	Contrast test in linear model	0.24	0.73
	other and tQ(UUG)C	effect difference	n = 22, n = 6 | df = 2728	Contrast test in linear model	2e-8	5e-7
	other and tQ(UUG)D1	effect difference	n = 22, n = 8 | df = 2728	Contrast test in linear model	0.35	0.78
	other and tQ(UUG)D3	effect difference	n = 22, n = 10 | df = 2728	Contrast test in linear model	0.48	0.84
	other and tQ(UUG)E1	effect difference	n = 22, n = 16 | df = 2728	Contrast test in linear model	0.36	0.78
	other and tQ(UUG)E2	effect difference	n = 22, n = 15 | df = 2728	Contrast test in linear model	0.86	0.97
	other and tQ(UUG)H	effect difference	n = 22, n = 3 | df = 2728	Contrast test in linear model	0.02	0.16
	other and tQ(UUG)L	effect difference	n = 22, n = 10 | df = 2728	Contrast test in linear model	0.49	0.84
	other and tR(ACG)D	effect difference	n = 22, n = 16 | df = 2728	Contrast test in linear model	0.40	0.82
	other and tR(ACG)E	effect difference	n = 22, n = 16 | df = 2728	Contrast test in linear model	0.38	0.81
	other and tR(ACG)J	effect difference	n = 22, n = 16 | df = 2728	Contrast test in linear model	0.13	0.58
	other and tR(ACG)K	effect difference	n = 22, n = 12 | df = 2728	Contrast test in linear model	0.59	0.89
	other and tR(ACG)L	effect difference	n = 22, n = 5 | df = 2728	Contrast test in linear model	0.33	0.78
	other and tR(ACG)O	effect difference	n = 22, n = 11 | df = 2728	Contrast test in linear model	0.69	0.94
	other and tR(CCG)L	effect difference	n = 22, n = 16 | df = 2728	Contrast test in linear model	0.43	0.82
	other and tR(CCU)J	effect difference	n = 22, n = 8 | df = 2728	Contrast test in linear model	0.99	0.99
	other and tR(UCU)D	effect difference	n = 22, n = 2 | df = 2728	Contrast test in linear model	0.20	0.68
	other and tR(UCU)E	effect difference	n = 22, n = 16 | df = 2728	Contrast test in linear model	0.67	0.94
	other and tR(UCU)G1	effect difference	n = 22, n = 12 | df = 2728	Contrast test in linear model	0.31	0.78
	other and tR(UCU)G2	effect difference	n = 22, n = 11 | df = 2728	Contrast test in linear model	0.88	0.97
	other and tR(UCU)K	effect difference	n = 22, n = 17 | df = 2728	Contrast test in linear model	0.89	0.97
	other and tR(UCU)M1	effect difference	n = 22, n = 18 | df = 2728	Contrast test in linear model	9e-11	4e-9
	other and tR(UCU)M2	effect difference	n = 22, n = 16 | df = 2728	Contrast test in linear model	0.65	0.92
	other and tS(AGA)A	effect difference	n = 22, n = 8 | df = 2728	Contrast test in linear model	0.32	0.78
	other and tS(AGA)B	effect difference	n = 22, n = 15 | df = 2728	Contrast test in linear model	0.16	0.64
	other and tS(AGA)D2	effect difference	n = 22, n = 17 | df = 2728	Contrast test in linear model	0.02	0.21
	other and tS(AGA)D3	effect difference	n = 22, n = 4 | df = 2728	Contrast test in linear model	0.40	0.82
	other and tS(AGA)E	effect difference	n = 22, n = 12 | df = 2728	Contrast test in linear model	0.12	0.57
	other and tS(AGA)G	effect difference	n = 22, n = 15 | df = 2728	Contrast test in linear model	0.10	0.53
	other and tS(AGA)H	effect difference	n = 22, n = 2 | df = 2728	Contrast test in linear model	0.05	0.39
S12 (continued)	other and tS(AGA)J	effect difference	n = 22, n = 16 | df = 2728	Contrast test in linear model	0.78	0.97
	other and tS(AGA)L	effect difference	n = 22, n = 18 | df = 2728	Contrast test in linear model	4e-7	8e-6
	other and tS(AGA)M	effect difference	n = 22, n = 13 | df = 2728	Contrast test in linear model	0.40	0.82
	other and tS(CGA)C	effect difference	n = 22, n = 12 | df = 2728	Contrast test in linear model	0.50	0.84
	other and tS(GCU)F	effect difference	n = 22, n = 20 | df = 2728	Contrast test in linear model	0.77	0.97
	other and tS(GCU)L	effect difference	n = 22, n = 15 | df = 2728	Contrast test in linear model	0.49	0.84
	other and tS(GCU)O	effect difference	n = 22, n = 16 | df = 2728	Contrast test in linear model	0.77	0.97
	other and tS(UGA)E	effect difference	n = 22, n = 6 | df = 2728	Contrast test in linear model	0.22	0.72
	other and tS(UGA)I	effect difference	n = 22, n = 9 | df = 2728	Contrast test in linear model	0.57	0.89
	other and tS(UGA)P	effect difference	n = 22, n = 11 | df = 2728	Contrast test in linear model	0.42	0.82
	other and tT(AGU)C	effect difference	n = 22, n = 10 | df = 2728	Contrast test in linear model	0.43	0.82
	other and tT(AGU)D	effect difference	n = 22, n = 4 | df = 2728	Contrast test in linear model	0.02	0.16
	other and tT(AGU)H	effect difference	n = 22, n = 5 | df = 2728	Contrast test in linear model	0.70	0.95
	other and tT(AGU)I1	effect difference	n = 22, n = 4 | df = 2728	Contrast test in linear model	0.87	0.97
	other and tT(AGU)I2	effect difference	n = 22, n = 10 | df = 2728	Contrast test in linear model	0.94	0.97
	other and tT(AGU)J	effect difference	n = 22, n = 16 | df = 2728	Contrast test in linear model	0.23	0.72
	other and tT(AGU)N2	effect difference	n = 22, n = 4 | df = 2728	Contrast test in linear model	0.09	0.53
	other and tT(AGU)O1	effect difference	n = 22, n = 1 | df = 2728	Contrast test in linear model	0.84	0.97
	other and tT(AGU)O2	effect difference	n = 22, n = 16 | df = 2728	Contrast test in linear model	0.52	0.86
	other and tT(CGU)K	effect difference	n = 22, n = 16 | df = 2728	Contrast test in linear model	0.78	0.97
	other and tT(UGU)G1	effect difference	n = 22, n = 10 | df = 2728	Contrast test in linear model	0.72	0.95
	other and tT(UGU)G2	effect difference	n = 22, n = 15 | df = 2728	Contrast test in linear model	0.63	0.92
	other and tT(UGU)H	effect difference	n = 22, n = 14 | df = 2728	Contrast test in linear model	0.45	0.82
	other and tT(UGU)P	effect difference	n = 22, n = 15 | df = 2728	Contrast test in linear model	0.35	0.78
	other and tV(AAC)E2	effect difference	n = 22, n = 9 | df = 2728	Contrast test in linear model	0.92	0.97
	other and tV(AAC)G1	effect difference	n = 22, n = 15 | df = 2728	Contrast test in linear model	0.36	0.78
	other and tV(AAC)G3	effect difference	n = 22, n = 19 | df = 2728	Contrast test in linear model	0.96	0.98
	other and tV(AAC)J	effect difference	n = 22, n = 9 | df = 2728	Contrast test in linear model	0.81	0.97
	other and tV(AAC)K1	effect difference	n = 22, n = 15 | df = 2728	Contrast test in linear model	0.34	0.78
	other and tV(AAC)K2	effect difference	n = 22, n = 17 | df = 2728	Contrast test in linear model	0.81	0.97
	other and tV(AAC)L	effect difference	n = 22, n = 14 | df = 2728	Contrast test in linear model	0.88	0.97
	other and tV(AAC)M1	effect difference	n = 22, n = 7 | df = 2728	Contrast test in linear model	0.45	0.82
	other and tV(AAC)M2	effect difference	n = 22, n = 14 | df = 2728	Contrast test in linear model	0.27	0.77
	other and tV(AAC)M3	effect difference	n = 22, n = 17 | df = 2728	Contrast test in linear model	0.25	0.74
	other and tV(AAC)O	effect difference	n = 22, n = 17 | df = 2728	Contrast test in linear model	0.75	0.96
	other and tV(CAC)D	effect difference	n = 22, n = 13 | df = 2728	Contrast test in linear model	0.34	0.78
	other and tV(CAC)H	effect difference	n = 22, n = 14 | df = 2728	Contrast test in linear model	0.93	0.97
	other and tV(UAC)B	effect difference	n = 22, n = 11 | df = 2728	Contrast test in linear model	2e-8	5e-7
	other and tV(UAC)D	effect difference	n = 22, n = 16 | df = 2728	Contrast test in linear model	0.43	0.82

Figure	Comparison	Alternative hypothesis	Sample size	Test	p-value	Adjusted p-value
S12 (continued)	other and tW(CCA)G1	effect difference	n = 22, n = 8 | df = 2728	Contrast test in linear model	0.02	0.16
	other and tW(CCA)G2	effect difference	n = 22, n = 11 | df = 2728	Contrast test in linear model	0.33	0.78
	other and tW(CCA)K	effect difference	n = 22, n = 5 | df = 2728	Contrast test in linear model	0.07	0.44
	other and tW(CCA)M	effect difference	n = 22, n = 19 | df = 2728	Contrast test in linear model	0.54	0.87
	other and tW(CCA)P	effect difference	n = 22, n = 15 | df = 2728	Contrast test in linear model	0.005	0.07
	other and tX(XXX)D	effect difference	n = 22, n = 18 | df = 2728	Contrast test in linear model	0.10	0.53
	other and tY(GUA)D	effect difference	n = 22, n = 18 | df = 2728	Contrast test in linear model	0.04	0.30
	other and tY(GUA)F2	effect difference	n = 22, n = 14 | df = 2728	Contrast test in linear model	0.32	0.78
	other and tY(GUA)J1	effect difference	n = 22, n = 3 | df = 2728	Contrast test in linear model	0.08	0.53
	other and tY(GUA)J2	effect difference	n = 22, n = 7 | df = 2728	Contrast test in linear model	0.21	0.69
	other and tY(GUA)M1	effect difference	n = 22, n = 17 | df = 2728	Contrast test in linear model	0.58	0.89
	other and tY(GUA)M2	effect difference	n = 22, n = 15 | df = 2728	Contrast test in linear model	0.47	0.83
	other and tY(GUA)O	effect difference	n = 22, n = 14 | df = 2728	Contrast test in linear model	0.11	0.55
