# Supplementary material for: Genome-wide mapping of individual replication fork velocities using nanopore sequencing
Source: Nat Commun. 2022 Jun 8;13:3295. doi: 10.1038/s41467-022-31012-0 (PMC9177527; doi:10.1038/s41467-022-31012-0)
Supplement: Supplementary file 2 — Description of Additional Supplementary Files [file 41467_2022_31012_MOESM2_ESM.pdf]

Description of Additional Supplementary Files

File Name: Supplementary Data 1
Description: Detailed information about the samples sequenced in this study.
