# Supplementary material for: Genome-wide mapping of individual replication fork velocities using nanopore sequencing
Source: Nat Commun. 2022 Jun 8;13:3295. doi: 10.1038/s41467-022-31012-0 (PMC9177527; doi:10.1038/s41467-022-31012-0)
Supplement: Supplementary file 4 — Reporting Summary [file 41467_2022_31012_MOESM4_ESM.pdf]

nature portfolio

Corresponding author(s): Laurent Lacroix, Olivier Hyrien, Benoît Le Tallec

Last updated by author(s): May 17, 2022

Reporting Summary

Nature Portfolio wishes to improve the reproducibility of the work that we publish. This form provides structure for consistency and transparency in reporting. For further information on Nature Portfolio policies, see our Editorial Policies and the Editorial Policy Checklist.

Statistics

For all statistical analyses, confirm that the following items are present in the figure legend, table legend, main text, or Methods section.

n/a	Confirmed	
☐	☒	The exact sample size (n) for each experimental group/condition, given as a discrete number and unit of measurement
☐	☒	A statement on whether measurements were taken from distinct samples or whether the same sample was measured repeatedly
☐	☒	The statistical test(s) used AND whether they are one- or two-sided<br>*Only common tests should be described solely by name; describe more complex techniques in the Methods section.*
☐	☒	A description of all covariates tested
☐	☒	A description of any assumptions or corrections, such as tests of normality and adjustment for multiple comparisons
☐	☒	A full description of the statistical parameters including central tendency (e.g. means) or other basic estimates (e.g. regression coefficient) AND variation (e.g. standard deviation) or associated estimates of uncertainty (e.g. confidence intervals)
☐	☒	For null hypothesis testing, the test statistic (e.g. F, t, r) with confidence intervals, effect sizes, degrees of freedom and P value noted<br>*Give P values as exact values whenever suitable.*
☒	☐	For Bayesian analysis, information on the choice of priors and Markov chain Monte Carlo settings
☒	☐	For hierarchical and complex designs, identification of the appropriate level for tests and full reporting of outcomes
☒	☐	Estimates of effect sizes (e.g. Cohen's d, Pearson's r), indicating how they were calculated

Our web collection on statistics for biologists contains articles on many of the points above.

Software and code

Policy information about availability of computer code

Data collection	MinKNOW (Oxford Nanopore Technology, MinKNOW Core versions 1.14.1 to 4.4.13) for MinION and PromethION sequencing data acquisition; CytExpert v2.4.0.28 for FACS data acquisition; ImageQuant LAS 4000 software version 1.3 for Western blot imaging.
Data analysis	R (versions 4.0.3 and 4.0.5); R packages kmlShape version 0.9.5, DescTools version 0.99.44, RcppRoll version 0.3.0, Hmisc version 4.6-0, tidyverse version 1.3.1, GenomicRanges version 1.40.0, rtracklayer version 1.48.0, BSgenome version 1.56.0, ggdist version 3.0.1, patchwork version 1.1.1, ggplot2 version 3.3.5, ggcorrplot version 0.1.3, ggpubr version 0.4.0, gridExtra version 2.3, modeest version 2.4.0, ggprism version 1.0.3, ggrepel version 0.9.1, furr version 0.2.3, devtools version 2.4.2, emmeans version 1.5.5-1, limma version 3.46.0; Samtools (version 1.10); Python (version 3.6); pomegranate Python package (version 0.14.4); Megalodon (Oxford Nanopore Technology, version 2.2.9); Taiyaki (Oxford Nanopore Technology, version 5.1.0); Guppy (Oxford Nanopore Technology, version 4.4.1); DNAscent (v1 and v2, custom software available in GitHub); RepNano (custom software available in GitHub); NanoForkSpeed (custom software deposited on GitHub); TraceFinder (Thermo Scientific, version 5.1); FlowJo v10.7.1; liftOver tool (http://hgdownload.soe.ucsc.edu/goldenPath/sacCer1/liftOver/).<br><br>Megalodon-based BrdU basecaller, NFS software and associated R scripts can be accessed at https://github.com/LacroixLaurent/NanoForkSpeed and https://doi.org/10.5281/zenodo.6535390. Custom R scripts for statistical analyses can be accessed at https://gitlab.pasteur.fr/gmillot/anova_contrasts/-/tree/v7.2.0. Python scripts are available at https://github.com/organic-chemistry/simunano.

For manuscripts utilizing custom algorithms or software that are central to the research but not yet described in published literature, software must be made available to editors and reviewers. We strongly encourage code deposition in a community repository (e.g. GitHub). See the Nature Portfolio guidelines for submitting code & software for further information.

Data

Policy information about availability of data

All manuscripts must include a data availability statement. This statement should provide the following information, where applicable:

- Accession codes, unique identifiers, or web links for publicly available datasets
- A description of any restrictions on data availability
- For clinical datasets or third party data, please ensure that the statement adheres to our policy

Nanopore sequencing data generated in this study have been deposited in the ENA database under accession code PRJEB50302 (https://www.ebi.ac.uk/ena/browser/view/PRJEB50302). Source data are provided with this paper or are available at https://github.com/LacroixLaurent/NanoForkSpeed and https://doi.org/10.5281/zenodo.5958270. Yeast genomic feature coordinates used in this study originate from UCSC SGD_other track (https://genome.ucsc.edu/cgi-bin/hgTables), oriented genes are from Ensembl database (Saccharomyces_cerevisiae.R64-1-1.104.gtf file downloaded from http://ftp.ensembl.org/pub/release-104/gtf/saccharomyces_cerevisiae/), replication origins are from reference 21 and replication timing data are from reference 42 (file GSM1036187_T7107_normalised.wig.gz downloaded from https://www.ncbi.nlm.nih.gov/geo/query/acc.cgi?acc=GSM1036187).

Field-specific reporting

Please select the one below that is the best fit for your research. If you are not sure, read the appropriate sections before making your selection.

☒ Life sciences ☐ Behavioural & social sciences ☐ Ecological, evolutionary & environmental sciences

For a reference copy of the document with all sections, see nature.com/documents/nr-reporting-summary-flat.pdf

Life sciences study design

All studies must disclose on these points even when the disclosure is negative.

Sample size	No sample size calculation was performed. Sample sizes range from a minimum of 2 to 22 for each experimental group or condition, with the rationale that every experimental condition/group be analysed independently at least twice. Additional studies were performed on over 125,000 individual replication fork velocities and on 10,000 to 100,000 simulated reads; these sample sizes were deemed sufficient to perform robust analyses.
Data exclusions	In all experiments involving fork speed estimation from nanopore sequencing reads, our signal treatment procedure, which includes data smoothing using a gaussian weighted rolling mean on 2.5 kb with exclusion of the first and last 2.5 kb windows, removed nanopore reads <5 kb; nanopore reads containing more than 2 rDNA repeats were also excluded in those experiments. Exclusion criteria were pre-established.
Replication	Replication fork speed at 25 and 30°C was estimated in WT-like growing yeast cells in 2 and 21 biological replicates, respectively, which all gave comparable results for a given temperature. All >125,000 fork velocity measurements from the 21 biological replicates of yeast cells grown at 30°C in standard conditions were pooled to (i) build the genome-wide map of replication fork progression, (ii) analyse fork speed at selected genomic features, (iii) compute a replication fork directionality profile, (iv) verify the spatial coincidence between known yeast origins and individual initiation sites. Experiments to estimate fork speed in mutant strains or in the presence of hydroxyurea were performed independently twice with similar results. Growth curves, FACS profiles and western blot analyses were all performed independently twice, with both experiments giving similar results. Development and validation experiments (basecaller comparison, test of BrdU incoporation in the MCM869, BT2 and BT3 strains, influence of BrdU concentration on fork speed estimation) were performed once.<br>All attemps at replication were successful.
Randomization	Randomization is not applicable to our study. Data shown herein are measurements of replication fork speed in wild-type or mutant yeast strains grown in various conditions. Samples therefore correspond per se to wild-type or mutant cells or to specific growth conditions and need not be further allocated into experimental groups.
Blinding	Blinding is not applicable to our study. As specified above, there was no group allocation. Data shown herein are measurements of replication fork speed in wild-type or mutant yeast strains grown in various conditions, which were analysed with the same parameters by an automated pipeline, "blind" by definition to samples' identity.

Reporting for specific materials, systems and methods

We require information from authors about some types of materials, experimental systems and methods used in many studies. Here, indicate whether each material, system or method listed is relevant to your study. If you are not sure if a list item applies to your research, read the appropriate section before selecting a response.

Materials & experimental systems

n/a	Involved in the study	
☐	☒	Antibodies
☒	☐	Eukaryotic cell lines
☒	☐	Palaeontology and archaeology
☒	☐	Animals and other organisms
☒	☐	Human research participants
☒	☐	Clinical data
☒	☐	Dual use research of concern

Methods

n/a	Involved in the study	
☒	☐	ChIP-seq
☐	☒	Flow cytometry
☒	☐	MRI-based neuroimaging

Antibodies

Antibodies used	Rad53 immunoblot was performed with rabbit anti-Rad53 antibody at 1:10,000 (Abcam #104232, batch GR3353005-2), using HRP-conjugated anti-rabbit (Promega #W401B) at 1:5,000 as secondary antibody.
Validation	From the manufacturer's website:<br>Product name: Anti-Rad53 antibody; Description: Rabbit polyclonal to Rad53; Host species: Rabbit; Tested applications: Suitable for: Western blot; Species reactivity: Reacts with: Saccharomyces cerevisiae; Immunogen: Synthetic peptide corresponding to Saccharomyces cerevisiae Rad53 aa 800 to the C-terminus conjugated to keyhole limpet haemocyanin.<br>References: ab104232 has been referenced in 51 publications.<br>Examples are listed below:<br>- Tannous EA et al. Mechanism of auto-inhibition and activation of Mec1ATR checkpoint kinase. Nat Struct Mol Biol 28:50-61 (2021).<br>- Johnson MC et al. Checkpoint inhibition of origin firing prevents inappropriate replication outside of S-phase. Elife 10:N/A (2021).<br>- Litwin I et al. Complex Mechanisms of Antimony Genotoxicity in Budding Yeast Involves Replication and Topoisomerase I-Associated DNA Lesions, Telomere Dysfunction and Inhibition of DNA Repair. Int J Mol Sci 22:N/A (2021).<br>- Koussa NC & Smith DJ Limiting DNA polymerase delta alters replication dynamics and leads to a dependence on checkpoint activation and recombination-mediated DNA repair. PLoS Genet 17:e1009322 (2021).<br>- Marsella A et al. Sae2 and Rif2 regulate MRX endonuclease activity at DNA double-strand breaks in opposite manners. Cell Rep 34:108906 (2021).

Flow Cytometry

Plots

Confirm that:

☒ The axis labels state the marker and fluorochrome used (e.g. CD4-FITC).

☒ The axis scales are clearly visible. Include numbers along axes only for bottom left plot of group (a 'group' is an analysis of identical markers).

☐ All plots are contour plots with outliers or pseudocolor plots.

☐ A numerical value for number of cells or percentage (with statistics) is provided.

Methodology

Sample preparation	Exponentially growing yeast cells were synchronized in G1 by addition of 0.2 µM α-factor for 3 hours then washed and resuspended in fresh, prewarmed medium containing 50 µg.mL-1 pronase to release them into the cell cycle. In the experiment examining the impact of BrdU on S phase progression, BrdU was added 15 min after cell release. Aliquots were taken at regular time intervals and fixed in ethanol. Fixed cells were washed with 50 mM sodium citrate pH 7.4, incubated in sodium citrate buffer supplemented with 0.25 mg.ml-1 RNAse A for 1 hour at 50°C, added with 2 mg.ml-1 proteinase K and incubated for one additional hour. DNA was counterstained overnight with SYTOX Green.
Instrument	Samples were analysed using a Beckman Coulter CytoFLEX LX flow cytometer.
Software	Data were collected using CytExpert v2.4.0.28 and analysed using FlowJo v10.7.1.
Cell population abundance	The whole cell population was analysed; no sorting was performed.
Gating strategy	Cells were first gated using the FSC-Area versus SSC-Area plot to remove debris, then interrogated by the ratios of area to height of the SYTOX Green signal (FL2-A versus FL2-H plot) to gate out cell doublets.

☒ Tick this box to confirm that a figure exemplifying the gating strategy is provided in the Supplementary Information.
