# Supplementary figures and images for: Genome-wide mapping of individual replication fork velocities using nanopore sequencing
Source: Nat Commun. 2022 Jun 8;13:3295. doi: 10.1038/s41467-022-31012-0 (PMC9177527; doi:10.1038/s41467-022-31012-0)

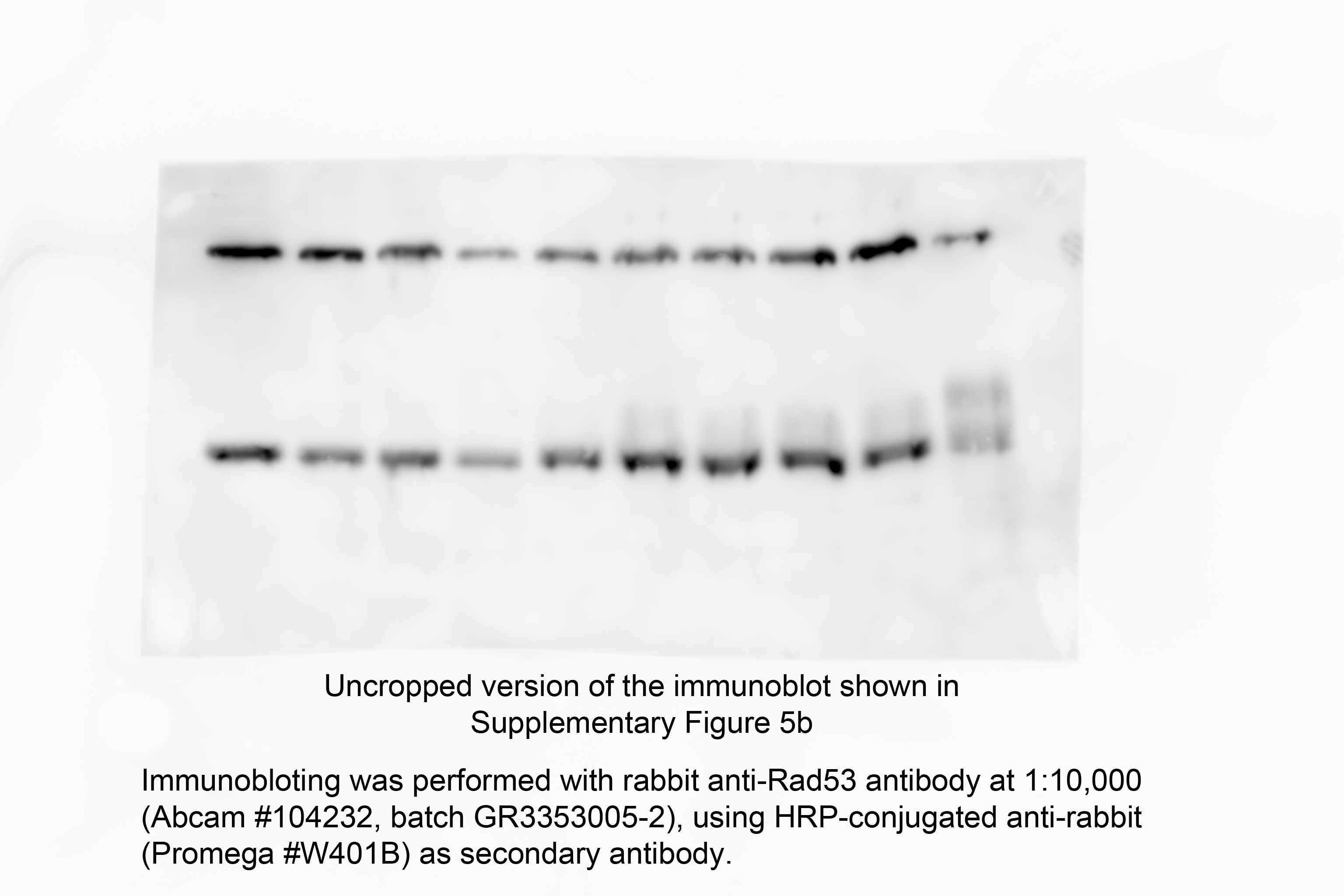

Supplement: Supplementary file 5 — Source Data [file 41467_2022_31012_MOESM5_ESM.zip › Source Data.tif]
